# Morph and Function: Exploring Origami-Inspired Structures in Versatile Robotics Systems

**DOI:** 10.3390/mi16091047

**Published:** 2025-09-13

**Authors:** Tran Vy Khanh Vo, Tan Kai Noel Quah, Li Ting Chua, King Ho Holden Li

**Affiliations:** School of Mechanical & Aerospace Engineering, Nanyang Technological University, Singapore 639798, Singapore; noel.quah@ntu.edu.sg (T.K.N.Q.); m230072@e.ntu.edu.sg (L.T.C.)

**Keywords:** origami-inspired robot (OIR), morphable mechanisms, foldable mechanisms, active origami, shape-morphing systems

## Abstract

The art of folding paper, named “origami”, has transformed from serving religious and cultural purposes to various educational and entertainment purposes in the modern world. Significantly, the fundamental folds and creases in origami, which enable the creation of 3D structures from a simple flat sheet with unique crease patterns, serve as a great inspiration in engineering applications such as deployable mechanisms for space exploration, self-folding structures for exoskeletons and surgical procedures, micro-grippers, energy absorption, and programmable robotic morphologies. Therefore, this paper will provide a systematic review of the state-of-the-art origami-inspired structures that have been adopted and exploited in robotics design and operation, called origami-inspired robots (OIRs). The advantages of the flexibility and adaptability of these folding mechanisms enable robots to achieve agile mobility and shape-shifting capabilities that are suited to diverse tasks. Furthermore, the inherent compliance structure, meaning that stiffness can be tuned from rigid to soft with different folding states, allows these robots to perform versatile functions, ranging from soft interactions to robust manipulation and a high-DOF system. In addition, the potential to simplify the fabrication and assembly processes, together with its integration into a wide range of actuation systems, further broadens its capabilities. However, these mechanisms increase the complexity in theoretical analysis and modelling, as well as posing a challenge in control algorithms when the robot’s DOF and reconfigurations are significantly increased. By leveraging the principles of folding and integrating actuation and design strategies, these robots can adapt their shapes, stiffness, and functionality to meet the demands of diverse tasks and environments, offering significant advantages over traditional rigid robots.

## 1. Introduction

### 1.1. The History of Origami

The Japanese art of paper folding, known as origami, has been practised for over a thousand years. Early folds were used for religious and ceremonial purposes in China and were subsequently improved in Japan [1]. For example, folded paper offerings (noshi) were given with gifts to represent good fortune and convey blessings, or Shinto rites were decorated with exquisite paper decorations that symbolised purity, protection, and the connection between the human world and the realm of spirits. Origami became a popular hobby among samurai and merchants during the Edo era (1603–1867). However, the techniques were mostly unwritten and were passed down through family or religious traditions.

In the mid-20th century, this classic Japanese art form emerged through the outstanding efforts of Akira Yoshizawa (1911–2005), who popularised origami through the publication of new models, exhibitions, and the creation of the International Origami Association [2]. This changed origami from a folk art to a discipline that people all over the world admire. His new methods of creating creases and utilising wet-folding techniques, which maintain sculpted curves on dampened paper, demonstrated that paper could produce smooth and realistic shapes. At the same time, Western mathematicians such as Humiaki Huzita (Japanese Italian) and Jacques Justin (French) began to develop the geometric laws that contribute to flat-foldability, now known as the Huzita–Hatori axioms [3] and Kawasaki’s and Maekawa’s theorems [4]. These rules provided a theoretical basis for the mechanical study of folds, thus establishing a new era in which origami has been propelled in engineering, led by the foundational contributions of exceptional individuals, including Koryo Miura, Robert J. Lang, Erik Demaine, Jacques Justin, and Tomohiro Tachi.

### 1.2. From Classic Origami to Engineering Applications

Since the 1970s, origami principles have started to be realised in engineering approaches, starting from deployable structures that utilised the Miura-Ori pattern developed by Koryo Miura, originally designed for quickly folding maps [5], to pack and deploy massive solar panels from small launch volumes. This parallelogram fold pattern, with a single degree of freedom (DOF), proved both mathematically elegant and practically indispensable for the compact storage and reliable deployment of large structures. Then, in the early 1980s, Kawasaki and Justin built a formal theory to determine the flat-foldability condition, which ensured that entire origami patterns could collapse without collision. Until now, this theorem has played an essential role in origami design. In the late 1990s, Robert J. Lang, a physicist and origami artist, revolutionised design by developing the Tree Method [6], an algorithmic approach that converts arbitrary three-dimensional shapes into flat crease patterns, thereby enabling systematic, computer-aided origami creation. In the early 2000s, Erik Demaine and collaborators, like Joseph O’Rourke and Martin Demaine, provided efficient algorithms and completeness results for testing rigid-foldability and flat-foldability, grounding origami’s kinematic behaviour in solid computational geometry. Finally, beginning in the late 2000s, Tomohiro Tachi translated these theoretical breakthroughs into practice with interactive simulation tools such as Freeform Origami [7] and thick-sheet modelling methods [8], allowing engineers to visualise, prototype, and fabricate complex foldable mechanisms with unprecedented fidelity.

These pioneering works have led to extensive research into new materials and designs inspired by origami for a wide range of applications, including biomedical engineering, robotics, architecture, and space exploration. Traditional papers in origami, which were normally used for prototyping, have been gradually replaced with metal foils, polymer films, and composite laminates for improved properties in various field requirements. Until now, this research trend in origami-inspired structures has been growing continuously, which proves how an ancient art form has evolved into a powerful engineering solution [9,10].

### 1.3. Significant Contributions of Origami-Inspired Structures in Robotics

Origami-inspired structures offer robotic systems numerous technical benefits by incorporating complex kinematics and adjustable mechanics directly into the robot’s body. Crease patterns function as built-in hinges and transmissions, eliminating the need for separate joints, linkages, and fasteners. This enables one actuator to produce large, multi-DOF movements with remarkable accuracy. Because folded substrates are naturally flexible, they can tune their stiffness and absorb shocks, making them safe for sensitive interactions yet strong enough to hold heavy loads when the folds are held in place. Additionally, being able to fold robots flat for travel and then use them independently reduces both the number of parts and the space envelope, which is crucial for deployable platforms in space. Finally, OIRs can function more effectively than traditional rigid designs because they can be easily integrated with sensors and actuators into the folding substrate. This makes them more functional and adaptable, which opens the door to agile manipulators, shape-shifting explorers, and wearable exoskeletons that can change to fit different tasks and dynamic environments.

With the increasing interest in adopting origami structures into engineering applications, several reviews have been released and updated over recent years to catch up with these novel design concepts. The most common domain in the literature reviews is toward targeting applications to which origami structures offer their unique advantages, such as biomedical applications [11], robotics [12], energy absorption [13,14], and space applications [15]. Design and optimisation methods design [16], mechanical characteristics, and modelling techniques [17,18] have also received many detailed reviews. Recent reviews are now focusing on updating the unique properties such as tunable stiffness and buckling from origami structures with their complex modelling techniques of buckling [19]. In addition, another fast-growing domain is toward the self-folding ability of origami-inspired structures that utilise the advantages of stimuli-responsive materials, a domain which has also been updated regularly over the past ten years [20,21,22]. Foreseeing the need for a comprehensive document, some good works have been published that cover the major factors, from crease patterns [23], to design, fabrication [12], and actuation techniques [24]. However, these studies are either outdated or emphasise particular domains for a focused review. Therefore, in this review, we provide a comprehensive and organised overview to systematically examine the full spectrum of OIRs, encompassing design, analysis tools, materials, fabrication, and actuation strategies across various scales. This review begins with the essential fundamentals of origami structures, equipping users with the core concepts to confidently apply these folding techniques in OIRs from Section 2 and Section 3. We then examine various materials and fabrication techniques, ranging from traditional cutting and manual folding to advanced manufacturing processes in Section 4. The actuation schemes are systematically categorised and reviewed in Section 5. More importantly, throughout the review, we highlight four key advantages and motivations of OIRs, in Section 3 and Section 5, that are as follows: (1) morphological capability, enabling on-demand shape and stiffness changes by the folding mechanism; (2) multi-DOF system, allowing the robot to make complex movements within a compact design of minimal actuators; (3) mechanical compliance, providing passive energy absorption and a safe human–machine interface; and (4) simplified deployable ability, allowing for reliable deployment while remaining compact for transportation or in idle conditions. Additionally, these applications are also categorised into scales, using which the advantages and limitations are revealed in Section 6. Finally, Section 7 presents a constructive discussion and outlook on the future of OIRs, culminating in a conclusion in Section 8. This well-organised review paper aims not only to provide comprehensive knowledge for researchers and engineers who want to start adopting this ancient art of folding in complex engineering challenges, but also to inspire origami artists and hobbyists on the engineering potential of origami beyond its true artistic forms.

## 2. Fundamental and Theoretical Background

### 2.1. Fundamentals of Origami

Origami involves the transformation of a two-dimensional (2D) planar sheet into a three-dimensional (3D) structure exclusively through folding operations, without cutting or stretching in its idealised form. In this section, we outline the fundamental components of origami structure and then describe the primary fold types and their corresponding characteristics.

#### 2.1.1. Anatomy of Origami

Crease patterns are 2D diagrams, which can be straight or curved, that design where and how the material will fold [25]. There are two fundamental folds in origami: valley and mountain. While the valley fold is the crease that folds inward, resembling a “valley” when viewed from above, the mountain fold folds outward, creating a “mountain” shape. These folds are colour-coded or represented with different line styles to guide the users. However, some complex crease patterns might require an expert level of knowledge or a simulation tool to be understood and followed successfully. In the engineering approach, creases can be viewed as mechanical hinges in the complete folded structures.

Besides the origami’s basic mountain and valley folds, which provide hinge action, there are complex crease folds that are primarily seen in artistic works, for example, swivel, inside/outside reverse, squash, and sink folds [26]. These folds are a combination of several pre-creases, eventually adding more details to the origami structure. For example, reverse folds usually enable the features such as limbs or beaks in animal designs, while the squash fold is a common technique to create new panels for detailed features, often seen in flowers [27]. In engineering applications, an effort was made to use the reverse-fold technique in the design of a bistable spherical four-bar mechanism [28]. However, these folds are not very popular due to the availability of suitable materials and fabrication techniques.

**Figure 1:**
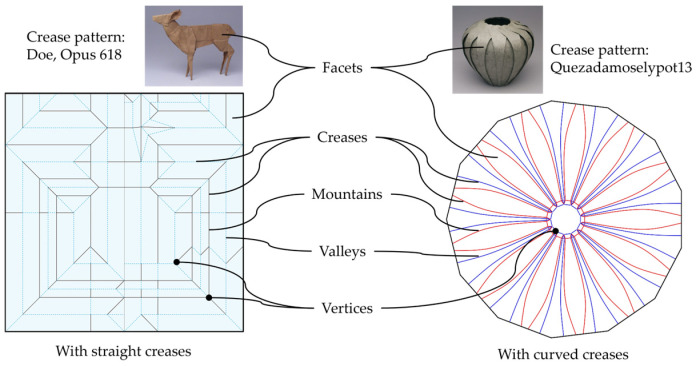
Illustration of the anatomy of origami with two crease patterns from Robert J. Lang’s origami website [27]. **Left**: Doe, Opus 618; **Right**: A pot, Quezadamoselypot13, Opus 710.

**Figure 2:**
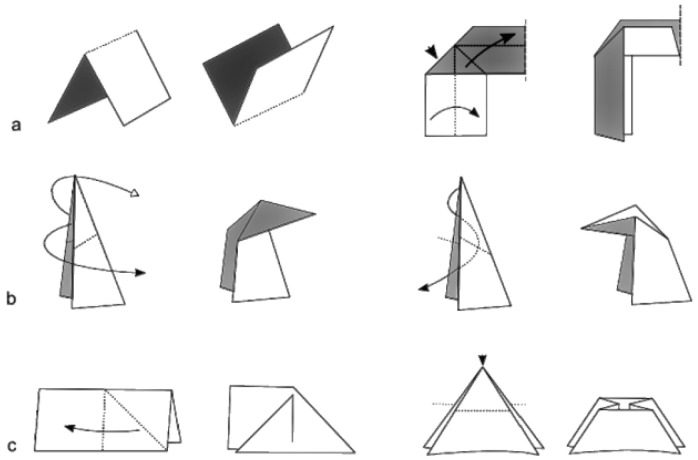
Fold types in origami. (**a**) Mountain, valley, swivel (two steps); (**b**) inside reverse (two steps), outside reverse (two steps); (**c**) squash (two steps), sink (two steps). Pre-print with permission from [26].

Facets are the flat panels bounded by creases. Traditionally, facets are assumed to be rigid, so all deformation occurs at the folds. However, in several engineering applications, facets can be deformed to act as an energy absorber [29]. A vertex is defined as a point where multiple creases meet, forming an angle or corner. The arrangement of crease angles around a vertex plays an important role in determining the final origami structure, whether it is flat-foldable or self-locking. This condition is realised by Maekawa and Kawasaki’s theorems, explained in the subsequent subsection. Figure 1 and Figure 2 below illustrate the anatomy and typical folds in origami.

#### 2.1.2. Crease Pattern Rules

**Figure 3:**
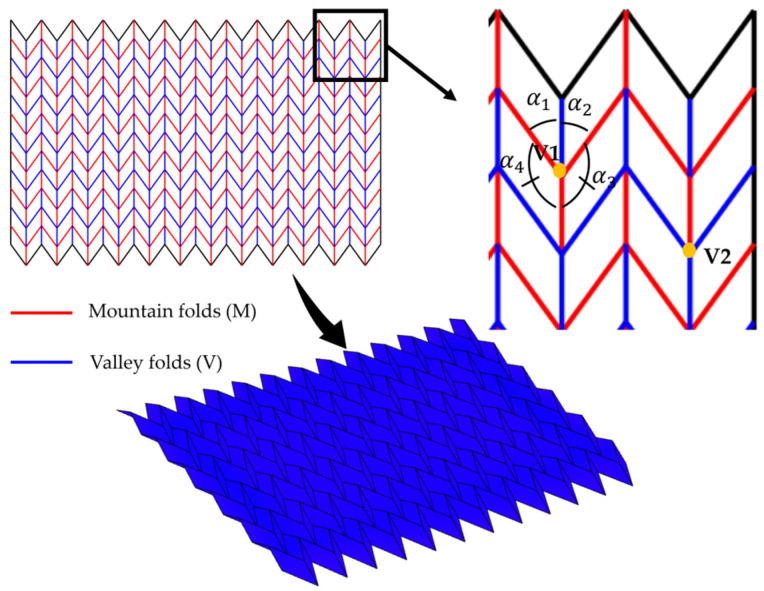
Illustration of Maekawa’s and Kawasaki’s theorems on a flat-foldable Miura-Ori.

There are two theorems that govern the crease rules. Maekawa’s (or Maekawa–Justin’s) theorem stated that at every vertex in a flat-folded origami, the difference between the number of mountain and valley creases is always two. Kawasaki’s theorem is a branch of the Maekawa theorem, which states that at any flat-foldable vertex, the sum of alternating angles must equal 180°. This ensures that there is no overlap or tearing when the model is pressed flat. Figure 3 below illustrates these theorems on a Miura-Ori design. Maekawa’s theorem is as follows: at vertex V1,

M − V = 3 − 1 = 2(1)

And at vertex V2,

M − V = 1 − 3 = −2(2)

And following Kawasaki’s theorem, at vertex V1, we have,

(3) $$\alpha_{1}+\alpha_{3}=180{}^{\circ},\;\alpha_{2}+\alpha_{4}=180{}^{\circ}$$

#### 2.1.3. Mechanism of Folds

To align understanding with the OIR design in the later stages, we classify the folding mechanisms into hinge-type or bending-type based on how the materials in the origami structure are involved in the folds.

A hinge-type fold, also known as a rigid or creased fold, is an ideal folding concept in which the material is sharply folded at the lines, and the creases act as perfect mechanical hinges. The facets are assumed to be rigid, thus remaining flat and undeformed. When an OIR design can be assumed to have no thickness, for example, in a paper-based prototype, precise folding can be achieved because the creases behave as perfect rotational hinges. Therefore, motion is considered to have high repeatability, with low or absent hysteresis.

In contrast, a bending-type fold, also known as an elastic/non-rigid fold or a smooth fold, involves a continuous and distributed bending mechanism of the facets. The creases now act as elastic zones, while the facets are capable of deformation under applied load. This fold is applied to those with thick facets and non-zero width creases (or flexible hinges). Therefore, this fold mechanism can generate a smooth or curved profile with a built-in compliant property.

### 2.2. Design Methods and Tools

The design of complex origami figures has evolved from an intuitive approach to an algorithmic approach [6]. Accurate computational models and simulation frameworks are essential for predicting the folding behaviour, mechanical performance, and control requirements of OIRs. Despite several papers reviewing the deep fundamentals of the mechanical characteristics of origami structures, the review of design approaches is merged with fabrication and applications, which can confuse readers regarding clear classifications of potential modelling methods [18,19,30]. This section provides a brief overview of different modelling approaches, along with an insightful discussion on their potential applications in OIRs.

#### 2.2.1. Kinematics-Based Methods

The kinematics-based approach focuses on the geometry and motion of the folds. By simplifying the complex behaviour of the origami structure, these models directly predict the shape change before and after the folds, hence making them suitable for applications such as foldable and deployable structures. In addition, they can be used for motion planning or fed into the inverse kinematics of the OIRs.

In kinematics-based models, the facets are assumed to be rigid and retain their shape, and deformation is only observed along the creases, similarly to hinge-type folding. Typical methods include the equivalent mechanism [31], direct geometric formulations from which the kinematics is derived from the mathematical relationships [32,33], and reduced-order models based on the symmetry of the system [18]. Kinematics-based models excel for motion planning, inverse kinematics, and real-time control when material deformations are negligible.

#### 2.2.2. Mechanics-Based Methods

The mechanics-based approach incorporates material properties into the models and considers the effects of forces, stresses, and energy in the folding process. Therefore, these models are suitable if the mechanical properties of the structure are needed. Typical approaches based on mechanics-based models are finite element-based and reduced-order models.

The first approach, or the finite element method (FEM), was used to investigate the stress and deformation of origami-inspired metamaterials under different load conditions to guide the design of the fold angle parameters and tune the compressive modulus for engineering applications [34].

The second approach can be seen in several models, such as the bar–hinge [35] and equivalent-truss model [36]. The origami structures, especially in 2D, can be analysed using the concept in the bar and hinge model. It models the structure as a network of rigid bars (facets) connected by rotational hinges. The axial stiffness property is assigned to the bar to simulate any shearing or deformation of the proper materials. Meanwhile, the rotational stiffness is applied to the hinges to simulate bending resistance, allowing for the folding to be realised. By doing so, it simplifies the complex origami structures and, to some extent, the properties of materials. This model was used to simulate effectively the essential behaviours of origami structure under stretching, shearing, and bending along the fold lines, as well as the bending of the facets [37]. Mechanics-based models are essential for structural design, material selection, and understanding force–deformation behaviour, load bearing, or energy storage.

#### 2.2.3. Hybrid Approaches

The methods mentioned above can be used together or in conjunction with other techniques to enhance the simulation efficiency for complex origami structures. In practice, these hybrid approaches often begin with a kinematic design for geometry and DOF analysis, followed by a mechanics-based analysis (via FEM or spring models) to validate strength, durability, and dynamic performance before fabrication. For example, a kinematic model with a parametric study was used to optimise the required bending motion of the origami finger with a Yoshimura-inspired design [38]. Similarly, an elasticity-based bar–hinge model with a combination of parametric studies was developed to guide the optimisation of Kresling-based unit frusta and their tunable stiffness [39]. FEM simulation was also used in the same work as a computational tool to verify the in-plane performance of the system against the results from the primary numerical model. In another work, a combination of an analytical model for the hard-magnetic soft material and an FEM approach for soft actuator’s deformation was used on a magnetic-driven actuator [40]. More importantly, a multiphysics simulation approach introduces heat transfer and thermomechanical coupling functions to the bar–hinge model via FEM and analytical simulations, effectively capturing the interdependent electro-thermal actuation of a Miura-Ori pattern [41].

#### 2.2.4. Quasi-Static and Dynamic Modelling Approaches

**Figure 4:**
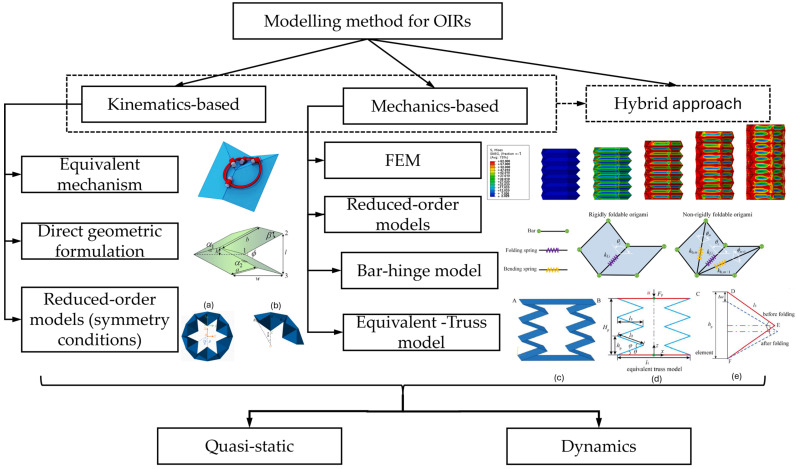
Diagram to present typical modelling methods for OIRs. Illustrations as examples for each method, as adapted from the literature. Kinematic-based methods: Equivalent mechanisms with a spherical four-bar mechanism for Miura-Ori [31]; geometrical model of unit cell Miura-Ori [32]; and reduced-order model from (**a**) a complete design to (**b**) 1/6 of a waterbomb design [18]. Mechanic-based methods: FEM for an origami bellows with Abaqus/Explicit [43]; bar–hinge model for rigidly foldable and non-rigidly foldable origami [19]; and (**c**) structure, (**d**) equivalent-truss model, and (**e**) geometry relation of the accordion origami [44].

The above models can be used for both quasi-static and dynamic modelling purposes. Moreover, it is essential to note that in complete origami structures, which comprise a series of folds, while some folds must be strictly folded one way in order to be valid, other designs, such as modular designs with repeated unit cells, can be folded through different processes. Even though the final structure could be identical, the dynamic behaviours could vary significantly. For example, a comprehensive analytical work from Ichiro and Masatoshi demonstrated that different initial geometries and folding patterns of the pantographic truss resulted in different dynamic loading capabilities [42]. In applications that require load-bearing capability, high stiffness, or impact load absorption, understanding both the static and dynamic behaviours of origami-based structures can effectively guide not only the geometrical design but also the folding sequences. Additionally, in OIR applications, the design of actuators, their integration strategy, and the efficient actuation paths can be revealed through proper simulation models. Figure 4 presents the classifications of modelling methods that are commonly used in origami structures.

#### 2.2.5. Tools for Origami Designs

**Figure 5:**
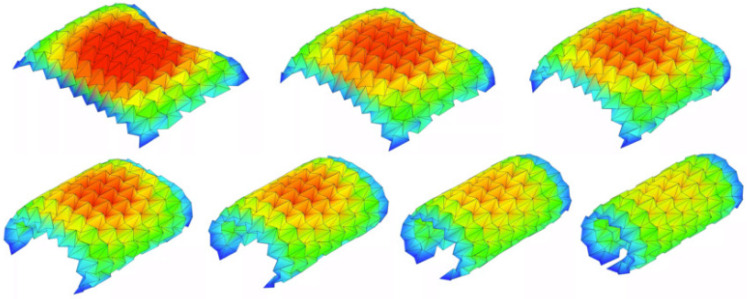
Example of strain visualisation of the materials under different folding states from the Origami Simulator [49]. The strain across the sheet is mapped to a colour code, from blue (no strain) to red (max strain).

**Figure 6:**
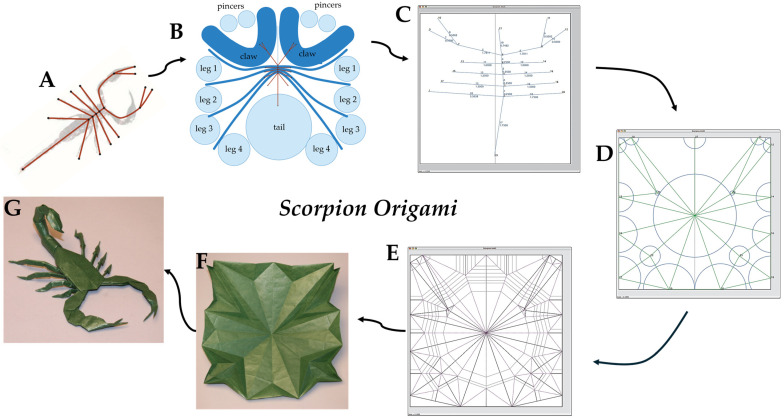
Illustration of a complete design process of an Origami Scorpion Varileg with TreeMarker software from Robert J. Lang, absorbed from [54]. (**A**) Stick figure over a scorpion photograph. (**B**) Stick figure transferring to circles and rivers to present minimum area of papers for each flap (features). (**C**) Stick figure on TreeMarker. (**D**) Optimisation with TreeMarker. (**E**) Final crease pattern on TreeMarker with mountain and valley folds assignment. (**F**) Folding on paper following the crease pattern. (**G**) The finished scorpion.

To accelerate design exploration, a suite of dedicated origami simulators has emerged that offer intuitive crease-pattern editors, real-time 3D folding visualisation, and collision detection, enabling designers to iteratively refine fold layouts without requiring custom coding. Through plugin architectures or export capabilities, these simulators can also directly work with CAD packages for OIR design. There are some algorithms and tools that allow people, from experts to hobbyists, to design complex origami crease patterns, such as TreeMarker [45], Origamizer [46,47], and Freeform Origami [7,48]. Additionally, Origami Simulator [49] is an open-source platform that simulates how an origami crease pattern will fold [50], utilising a solver based on the works of Tachi [51], Mark and Simon [52], and Kosuke and Jun [53]. Figure 5 below illustrates an example of the strain across the origami sheet in various folding states from the Origami Simulator. This tool allows the users to input the strain threshold to examine how much the sheet can be deformed under load conditions. This maximum strain will appear as the red colour for visualisation. This strain visualisation profile will change according to the user’s setup, assisting them in primarily justifying the potential mechanical properties of the selected model. Additionally, an overview of the process for transferring an idea into a final origami scorpion using TreeMarker software version 5.0.1 is illustrated in Figure 6.

## 3. Design of Origami-Inspired Robot (OIR)

### 3.1. Design Methodologies

#### 3.1.1. Traditional Tessellation Patterns

**Figure 7:**
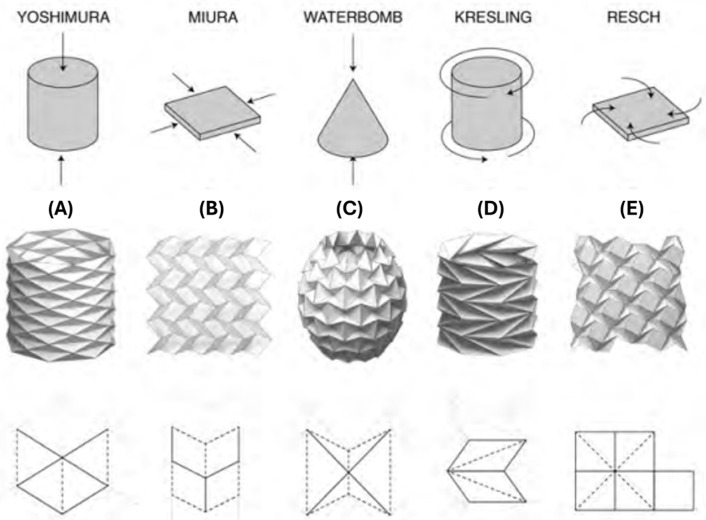
Demonstration of the geometric relationship between the force and folding patterns of typical tessellation designs. **First row**: force equivalent mode; **second row**: folding geometry; and **last row**: crease pattern [61]. The equivalent force in tessellation designs: (**A**) Yoshimura: cylindrical compression; (**B**) Miura: transverse planar compression; (**C**) Waterbomb: conical compression; (**D**) Kresling: rotational-twist-compression of a cylinder; and (**E**) Resch: torsional compression of the plane.

Tessellations are the most common patterns that have been adopted for engineering applications. They can be categorised as flat patterns, which Miura [55] and Resch patterns [56] are considered to be, and tubular and arched patterns, which include Kresling [57], Yoshimura [58], accordion [59], and waterbomb patterns [60]. Typical tessellation patterns are shown in Figure 7.

The Miura-Ori pattern consists of repeating parallelogram units joined along alternating mountain and valley folds. Because the entire sheet expands or contracts in a single degree of freedom, Miura-Ori has been widely adopted for deployable robotic skins, grippers that wrap around objects, and compact booms that stow flat and unfurl smoothly. Its uniform kinematics and linear expansion ratio make it easy to model and control.

The Resch (or Ron Resch) pattern is composed of repeating quadrilateral cells arranged such that opposing creases form a network of alternating mountain and valley folds, but offset to produce a twisting, deployable sheet. Under transverse compression, the pattern shears uniformly, allowing the entire panel to contract in one direction while simultaneously expanding in the perpendicular direction. Similarly to Miura-Ori, the Resch pattern’s homogeneous kinematics and linear shear ratio make it straightforward to model, predict, and control in robotic applications.

The Kresling pattern forms a series of triangular panels arranged in a ring. In 3D, it appears as a chiral tower with a polygonal base that expands and contracts through coupled longitudinal and rotational motion. It offers tailorable multi-stability and tunable stiffness. While generally not rigidly foldable, its high compressibility and fewer folds can enhance durability.

The Yoshimura pattern is a cylindrical crease pattern that generates a purely translational motion element. It is noted for its high folding efficiency and is often considered rigid-foldable, though panels may deform under axial folding in real materials. This pattern can induce controllable buckling modes, applied to morphing robots that switch between stiff and compliant cylindrical shells. The accordion pattern is a planar-based design that mimics the accordion music instrument. However, in engineering applications, the accordion pattern usually appears in a tubular shape and produces the axial compression movement similar to the Yoshimura design.

The waterbomb pattern is built from an array of diamond-shaped panels that form a four-vertex pattern. When compressed, the sheet transforms from a flat layout into a three-dimensional, dome-like structure and can snap back again, exhibiting a bistable, bellows-like behaviour.

#### 3.1.2. Single-Vertex Patterns

Single-vertex patterns feature crease networks that radiate from a central hinge, producing a radial folding. By arranging crease lines around a central vertex, similar to a petal figure, this structure facilitates the unfolding of the end-effectors, such as those found in circular grippers [62], the folding wings of insects or birds [63], or space deployable–foldable truss-antenna mechanisms [64], with minimal actuation complexity. Common patterns in this group are petal and flasher patterns. The petal pattern resembles flower petals radiating from a central vertex. In engineering applications, it usually appears as multiple triangular panels or frames that radiate and fold inward [64]. The flasher pattern comprises radial spirals of alternating crease lengths, enabling rapid, symmetric contraction to a compact form [65]. They exhibit remarkable rotational symmetry and a high expansion-to-compression ratio. Its circular design helps avoid uneven deformation when materials are not rigid enough.

#### 3.1.3. Modular Unit Cells Design

Modular unit cell design refers to the design in which the unit module can be repeated to achieve various geometries and properties. The tessellation and single-vertex patterns introduced above are typical designs following this approach. Modular unit cells can tile arbitrarily on a large scale and can be programmed to tune stiffness and shape both locally and globally. Each cell can be parameterised by folding angle, panel aspect ratio, or hinge stiffness, so that global shape and stiffness gradients emerge from simple local rules [16,18,19,66]. However, rather than enforcing strict periodicity, the modular unit can also be optimised for a particular local function or load case across the structure. This functionally graded approach can produce curved morphologies, spatial stiffness gradients, or region-specific behaviours such as bistability or compliance. Modular design is not limited to 2D crease patterns or unit cell folds, but also occurs in 3D modules. For example, a universal module based on Kresling design and inflatable cells can generate up to seven motion modes while being actuated by a pneumatic actuation [67].

#### 3.1.4. Custom Crease Design

Custom creases can be designed with the help of computational tools, as reviewed in Section 2.2.5, or by imagination and experience, to achieve unique kinematic and load requirements. Custom patterns have produced origami-inspired robotic arms that mimic human-like joint sequences, metamorphosis robots, and deployable antenna arrays with non-uniform expansion.

By combining these foundational methodologies and drawing on emerging patterns, researchers can engineer OIRs to achieve complex shape changes, multimodal compliance, and scalable deployment across a spectrum of applications.

### 3.2. Designs Toward Applications

#### 3.2.1. Morphological Capability

Origami-inspired structures offer a powerful mechanism to equip robots with on-demand shape-shifting capabilities. With the crease patterns, complex actions through various combinations of bending, twisting, contraction, and extension, can be realised [52], thus resulting in a morphable system. Several origami patterns have been investigated and adopted in engineering design to achieve a morphable property. The key design factors rely on the core folding geometry–force relation, as shown in Figure 7. For example, a single-DOF expansion of the planar Miura-Ori could be curved to form an adjustable wheel in OriWheelBot to seamlessly negotiate obstacles and adapt to the hardness of different terrains [68]. A similar design approach for morphable wheels was employed by other robots, which utilised the spiral fold of the vertex-centre pattern [69,70] and waterbomb patterns [71] to aid their mobility and adapt to various terrains and paths. In addition, a swift shape-shifting property can also occur in bistable or multi-stable mechanism designs, such as those from single-vertex unit [72], Miura-Ori-based [73], Kresling-based [36], and waterbomb-based [74] designs, or even by the reverse-fold concept [28]. Origami custom design can also be used to morph and reconfigure the robot for multi-tasks, including waking, sailing, rolling, and gliding [75]. For example, a multigait Tribot with embedded sensors and actuators at the hinge can reshape to stand, crawl, and jump [76].

Besides mobility capability, these morphable structures can also be employed in engineering design concepts to achieve multifunctionality that would be difficult or impossible with traditional rigid-body structures, which are multi-DOF motion, mechanical compliance, and simplified deployment across application scales and domains.

#### 3.2.2. Multi-Degree-of-Freedom (DOF) Motion

Multi-DOF motion is one of the essential advantages derived from the morphological capability of origami-inspired structures. Take an example of the universal module from Kresling-adopted patterns coupled with the inflatable feature, by joining three modules and being activated in different modes, three unique flexible motions were effectively demonstrated [67]. In addition, the modular design approach also allows for freedom in the number of modules that can be stacked to reach the required motion ranges. Another robot design with a Kresling structure can generate bending motion while standing alone, but allows complex motion, including six DOF crawling movement with stacking units [77]. A cylindrical Yoshimura structure is another typical design employed in multi-DOF applications such as robotic arms, pipe robots, and soft grippers [78].

As the movement and performance abilities of origami structures are obtained by their natural folds, they can perform in their designed multi-DOF manner with minimal actuations that activate their folds. The tendon-driven mechanism is an excellent example of this advantage, which we discussed later in Section 5.1.2. This feature favours the compact and lightweight design of the OIRs over their rigid body counterparts, in which the actuators or their motion and load transmission systems are needed at every joint. In comparison to soft robots with multi-DOF designs, in which the load capability relies on the properties of soft active materials [79,80], OIRs could possibly bear higher loads thanks to their rigid facets. Therefore, multi-DOF designs based on soft systems are more favoured for sensor applications [81,82,83].

#### 3.2.3. Mechanical Compliance

The foldable and morphable abilities of the tubular origami structures also lead to the compliant properties that mean it can function as a “soft” gripper. Several hard–soft coupling designs utilising the Yoshimura pattern [38] and waterbomb pattern [84] for compliant grippers have successfully demonstrated their potential for picking up objects of various shapes. In addition, tessellation patterns such as Miura-Ori and Ron Resch structures possess a unique geometry-induced negative Poisson’s ratio, so they are perfect choices for applications involving passive energy absorption and protection under impact and high-load conditions [13,19]. Honeycomb is another origami-inspired structure that has demonstrated its energy-absorption characteristics, which have been numerically proven [29]. In addition, several origami-inspired structures, both traditional tessellation and custom cutout designs, could be used in those applications that utilise the stiffness-tunable properties. For example, the custom diamond cutout design could be used with embedded force sensors that sense the applied pressure-induced variable stiffness to facilitate monitoring and adaptation for safe robot–human interaction [85]. The Kresling patterns are most commonly designed to be adopted for frusta-corrugated tubes with high-DOF tunable axial and bending stiffness [39,86].

#### 3.2.4. Simplified Deployment

Deployable mechanisms are one of the important applications of origami structure, since its very first applications to deploy solar panels with the Miura-Ori design [5]. This single-DOF planar tessellation structure promotes a smooth unfold with a single driving point for both length and width simultaneously, due to the unique parallelogram geometry, but also allows for compact transportation. The flat-folded structures are adapted to the deployable mechanism to ensure a smooth deployment process without interference, minimising the risk of deployment jams. Furthermore, those traditional tubular tessellation structures can be stiffened for load-bearing applications, making them suitable for deployable shelters and bridges [87]. Besides tessellation patterns, patterns with a central vertex, such as multi-petal- or flasher-based, have also demonstrated remarkable benefits for deployable structures, such as space arrays and solar panels [70]. Their circular-oriented designs help avoid uneven deformation to aid in a smooth and uniform deployment stage [65]. Besides those large-scale applications for space exploration, deployable origami structures are used in biomedical applications [11], in which the tubular tessellation accordion fold has been used as an origami stent [88].

**Figure 8:**
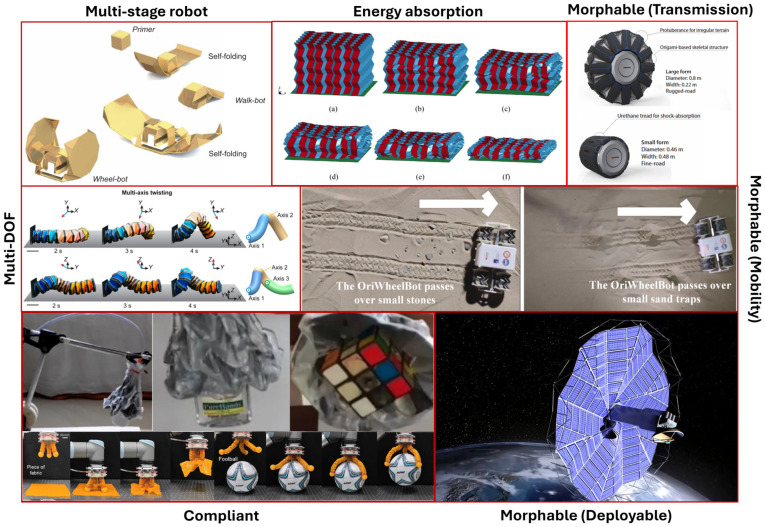
Typical functions from morphable origami-inspired structures. **First row** (**from left to right**): multi-stage robot using origami exoskeletons [75], out-of-plant deformation process from (**a**–**f**) for energy-absorption application using origami honeycomb from original stage to fully deformed stage [29], and high-load capacity transformable mechanism with membrane origami wheel [89]. **Second row**: Omnidirectional motions from Kresling-based robotic arm [90] and origami-wheel with adjustable width for sand walking versatility [68]. **Third row**: waterbomb-based gripper [84] and Yoshimura-based gripper grasping a piece of fabric and a football [91], and vertex-central-based solar panel mid-deployment (rendition) [92].

Figure 8 summarises the key function domains of morphable origami-inspired structures, while Table 1 maps these typical applications to the design methods and origami patterns, providing a clear picture of the morph-function capability of origami structures.

## 4. Fabrication Techniques for Origami-Inspired Robotics

### 4.1. Materials Selection

Material plays a crucial role not only in determining the final structure’s mechanical properties through facets and hinges, but also in selecting the fabrication and assembly processes.

The development of origami begins with paper and paper laminates. For instance, locomotion robots are made up of folded papers to mimic the soft-bodied, flexible, and smooth movement and motion of earthworms, which are ideal for search and rescue missions. Key performance indicators include achieving high axial and radial deformation ratios, as well as the implementation of a motor, which enables fast actuation and durability [98]. Another example of origami-inspired application is via the cut-fold process to mimic Venus flytraps’ behaviour by implementing an origami multiplexed switch (OMS) fabricated from bistable beams and conductive resistive actuators, which offers fast and controllable action [99] and enables the robots to act like a logic gate and/or an electrical switch, which allows it to autonomously detect and respond [100]. Progressively, the strength of papers used in OIRs has been further enhanced to adapt to the requirements through various techniques, such as coating or reinforcement. A prototype of a wheel robot is made from paper with edges reinforced using Carbon Fibre Reinforced Polymer (CFRP) and silicon polymer Ecoflex for friction enhancement [70]. Two plate springs are also embedded in the wheel to maintain its stiffness and allow it to recover after deformation. Additionally, paper coated with polyimide film (Kapton) was used for both the facets and creases [71] to be easy to fold but also showed higher resistance to shear stress.

Polymer films such as PET, polyimide, and Mylar have been widely used [101] as they offer high-temperature stability, as seen from the glass transition temperature of Kayton-type films, which significantly increases beyond 520 °C [102], and desirable mechanical and electrical properties [101]. The common material for fabricating flexible hinge joints is Kapton Polyimide [103]. Such properties enable repetitive foldable and joint-like movement with minimal material fatigue [104].

Composites and metal foils are also commonly used in those prototypes that require higher performance. For example, silicone–paper composites have higher reliability than paper and are used to mimic Iguana skin for robots’ resilience and toughness against repetitive dynamic forces [105]. Composite and multi-material hinges to enhance the load and fatigue resistance capabilities of the hinges [106]. Metals are valued in self-folding origami due to their properties, such as electrical conductivity. Metal offers better conductivity as compared to polymer composites when integrated with electrical control systems [107]. In some applications, a metal sheet is composited with soft materials to form a robust yet flexible design. For instance, the OrirWheelBot uses titanium alloy sheets as facets and high-viscosity flexible material—mesh fibre tape—for the creases [68].

Another group of materials that has received high attention in origami-inspired structures research, known as smart or active materials for actuation and sensor purposes, will be reviewed in detail in Section 5.3.

### 4.2. Fabrication and Folding Methods

Fabrication methods for origami structures can be classified into two categories: subtractive and non-subtractive methods. Traditional subtractive methods can be listed as laser etching and CNC (computer numerical control) cutting for metal foils and composites, or a manual scraper, mainly for low-strength materials like papers and reinforced papers. Cutting and patterning are the foundation of fabricating OIRs. Laser machining is effective on a wide range of materials. In several existing works [98,101,103,108], CO_2_ is used for scoring due to its low power and high speed, which reduces the thickness of the materials and creates flexible hinges. Laser cutting can be used to cut out shapes and patterns which are difficult to achieve via traditional fabrication methods [109]. As origami structures are mainly fabricated in thin-sheet materials, the machinery processes use very low power laser settings to engrave rather than cut, to etch the crease patterns [24,71].

Non-subtractive methods include laminating, bonding, and moulding or stamping. Lamination involves bonding multiple layers of dissimilar materials, such as flexible films wrapped around hard facets, to create robust creases and durable structures [36]. In a similar manner, bonding methods are used for different layers of facets, such as in titanium alloy sheets and flexible 3D-printed creases from mesh fibre tape for a Miura-inspired wheel, for example [110]. Once the crease patterns are produced, the folds can be performed manually by pressing sheets between dies or melting base materials into a mould, so-called “foldcores” [110]. However, some folds can be performed using thermal stimulus, so-called “fold and bake” to achieve the designed shape [111]. In designs with a thick structure, the method to create perforating crease lines has been widely used to reduce the stiffness along the folds, hence enhancing the fatigue performance of the overall origami structure [112,113].

### 4.3. Advanced Manufacturing Techniques

Advancements in additive manufacturing (AM) are revolutionising origami fabrication by enabling complex geometries, multi-material integration, and durability enhancement. Fused Deposition Modelling (FDM) printing is the most common method, using single to multi-materials, both locally and globally, for the structure. For example, the entire prototype of tessellated metamaterials [34] or multiloop origami-inspired spherical wrist mechanisms [31] can be 3D printed with PLA (polylactic acid). In another application, the 3D-printing method is only used to create the creases from mesh fibre tape (high-viscosity flexible material) [68]. Multi-material printing can be achieved, for example, with LA6 and Aramid fibres in a PA 6 matrix for a reinforced hinge [106]. In applications where soft–hard coupling performance is required, soft TPU (Thermoplastic polyurethane) and hard PLA are both printed for crease and facets, respectively, in a flexible robotic gripper [38]. The other AM methods, although not as popular as FDM, have also been utilised to manufacture the origami structure for different types of materials. For instance, Polyjet printing was used to jet photopolymer droplets and cure them with UV light, allowing for seamless multi-material integration and functional structures [12]. This was used for creating active composite hinges with shape memory behaviours. Additionally, multi-materials, including flexible photopolymer, rigid photopolymer, and support material, were inkjet-printed simultaneously for a polymer hinge [106].

While 3D printing offers high precision and design flexibility, traditional methods, such as manual folding, are still prevalent, especially for prototypes, due to their low cost and speed. When mass manufacturing is being considered in the future, technical solutions to achieve high repeatability and enhance the fatigue performance at creases/folds should be considered.

## 5. Actuation and Control Strategies

Form factor in origami-inspired robots is one of the key considerations in design. This impacts mobility, strength, and fabrication, and thus should be carefully considered. The form factor design in origami-inspired robots, while flexible, has been functionally limited to specific actuation control methodologies. While there are limitless possibilities and shapes origami-inspired robots have to offer, the current literature informs us that there is much to be explored. This paper identified and categorised these actuation modes as mechanical, fluidic, and embedded materials, as listed with typical actuation types in Table 2.

Actuation and control strategies in robots vary depending on their functional requirements and target applications. In robotics, one method of classification would be to distinguish between soft and hard actuation types. Compliant and deformable materials characterise soft actuation, whereas hard actuation is used to describe the traditionally known actuation methods via rigid components like gears, motors, and hydraulics [68,114,165,166,167]. Recent studies aim to reconcile the distinctions between soft and hard actuations through hybrid systems that integrate the advantages of both properties, merging the flexibility and adaptability of soft materials with the strength and control of rigid components to better emulate biomimetic movements [40,132,167,168]. This characterisation extends into origami-inspired robots, whose advantages, such as compactness and deployability, material and fabrication simplicity, and scalability with multifunctionality, have generated growing interest in fields like medicine [88,115,145,169], underwater and space deployments [68,114,166], and search-and-rescue [158,170].

The flexibility of OIRs with different form factors and actuation modes allow for unique control strategies. The multimodal actuation potential also requires controllers to adapt to large geometric deformations, varying stiffness profiles, or nonlinear coupling. Unlike rigid body systems, origami robots can consist of compliant hinges, foldable laminates, or cable-driven transmissions that depend on varying degrees of freedom and motion dynamics. As a result, compilation of control strategies is not quite classifiable due to its wide range of application dependencies [150,171,172].

For example, Yang et al. [173] developed an origami-inspired drone that folds and extends via a motor-driven origami mechanism, which alters the moment of inertia (MoI) and aerodynamics. Hence, the control inputs are highly dependent on factoring modified MoI in translational and rotational control. Alternatively, Zhakypov et al. [171] introduces a Tribot, built with quasi-2D sheets of material, that enables two locomotion modes: crawling and jumping, which are governed by second-order linear motion and a mass-spring-damper system, respectively. Li et al. [150] developed a model using dielectric elastomer folding actuators that implements two main methods of actuation: multilayer bending, and minimum energy structure. The former method utilises voltage to induce bending, and the latter takes advantage of potential energy in pre-stretched dielectric elastomers to manipulate joints by the change in angle.

The above examples are complex models that consider design and actuation types to derive control mechanics. These models can be distinguished independently through design methods (as discussed in Section 2.2) and actuation types. The following sections consider the actuation types and give a brief description on the control methodologies.

### 5.1. Mechanical Actuation

In this paper, we define mechanical actuation as the use of a power-driven technique to incur movement or functionality. In origami-inspired robots, there are generally three different subtypes: motors, tendons, and bistable snap mechanisms. Direct drive motors tend to exhibit higher torque and precise control, which enables robots to bear higher loads. Using tendons or linkage approaches enables mechanical actuation to operate along creases or folds. A bistable snap mechanism is applied when an application requires state stability.

#### 5.1.1. Motors

**Figure 9:**
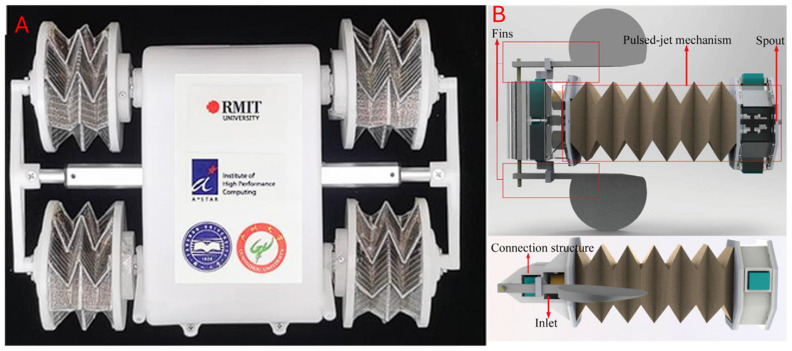
(**A**) An extensible-wheeled origami robot that uses motors to expand or contract, allowing for a better grip [68]. (**B**) A squid-inspired robot that can swim with a pulse-jet mechanism driven by a DC motor [174].

Here, we define ‘motors’ as embedded drivers that directly connect to any linkages, joints, or fold lines. Direct drive motors can be configured with high torque and precise control and are typically controlled with voltage or current. This usually allows for better folding actuation in terms of power and sensitivity, as mechanical reductions, such as gearboxes and pulleys, are minimised. However, this comes at the cost of complex system-wide integration, resulting in greater structural rigidity and overall weight. Origami-inspired robots are favoured for their flexibility; hence, direct drive motors are not usually preferred. Liu et al. [68] developed an origami-wheeled robot that has extendable wheels to overcome challenging terrains. The origami grooves integrated into the wheel as functional and folding anchors, helping the vehicle maintain traction on loose terrain during rotation. In a similar fashion, an extensible body was developed by Yang et al. [114], where the origami creases were used as fins to assist in propulsion for swimming. Figure 9 shows examples of motor-based actuation.

#### 5.1.2. Tendon/Cable-Driven

**Figure 10:**
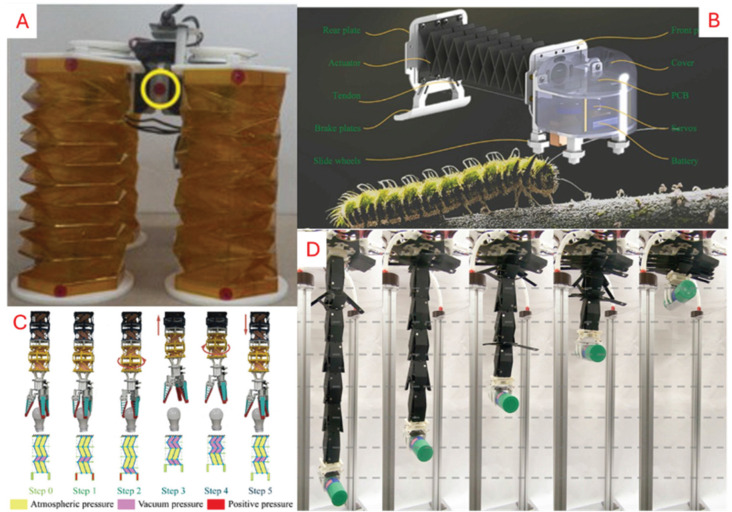
(**A**) Tendons to extend Kresling folded legs [119]. (**B**) Crawling-caterpillar-inspired tendon-based robot [175]. (**C**) Trunk-like continuum robot twisting a light bulb in one cycle with pneumatics and cable-driven mobility [125]. (**D**) Cable-driven extensible grasper that has bistability-snap mechanisms governed by magnets [122].

Tendon-based actuation transmits force through tensioned cables to generate movement or folds in origami structures. Tendon-based actuation considers tendon length and tension as control mechanisms. Unlike its motorised counterpart, incorporating a cable-driven setup usually leads to a lightweight configuration with easier integration due to a centralised controller layout. A functional setup for tendon utilisation can be seen in Figure 9, where Kim S. et al. [122] developed a foldable self-locking robot arm to allow for picking and placing objects with a drone. This robot arm design also highlights the modular potential enabled by tendon-driven actuation systems. Alternatively, Kim J. et al. [119] developed a quadrupedal tendon-driven walking robot that enables complex range and motions. The authors noted that the robot also exhibits shock absorption during traversal due to its compressible and bendable compliance. Other examples can also be seen in Figure 10.

#### 5.1.3. Bistable Snap Mechanism

**Figure 11:**
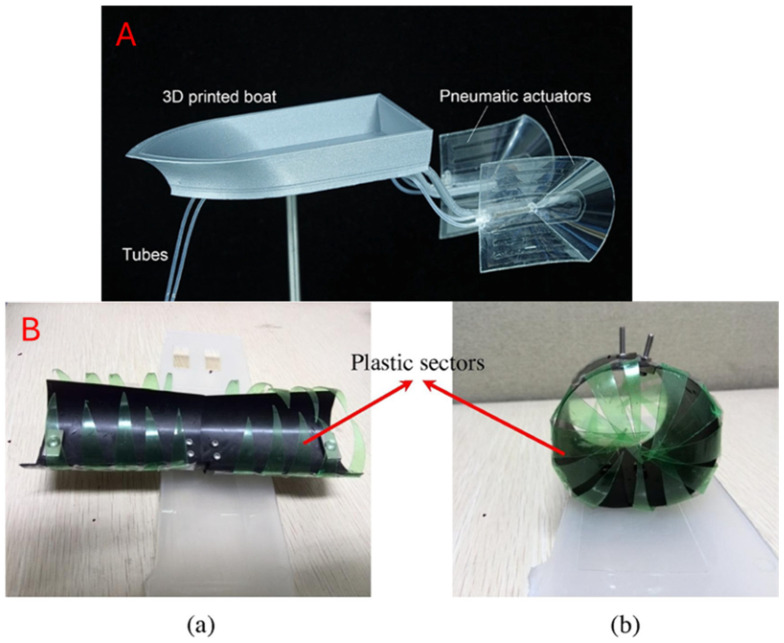
(**A**) A pneumatic actuator that operated with bistable snap propellers [176]. (**B**) A bistable Venus flytrap origami robot that activates with magnetic actuation at (**a**) opening stage, and (**b**) closing state [177].

A bistable snap mechanism refers to a structure with two discrete and stable states. Origami-inspired designs possess the flexibility to achieve bistability with specific crease or fold patterns [126]. The design space typically incorporates buckling or prestressed locking mechanisms that can be mechanically activated to transition between the stable states. This may not be reversible and can be designed for one-time use, utilising lock mechanisms that rely on mechanical rupture, thermal degradation, or plastic deformation. The typical control strategy includes a trigger pulse of force, pressure, or heat. One such example can be seen in Buscicchio et al. [129], who developed nanosatellites with a lightweight and compact solar panel that incorporated a deployable design. The origami configuration allowed for efficient packing during launch and expanded in orbit to maximise sunlight exposure. Liu et al. [121] developed a bistable gripper mechanism that also enabled precise pick-and-place operations, demonstrating operational feasibility. Figure 11 displays some examples of bistable snap mechanisms.

### 5.2. Fluidic Actuation

**Figure 12:**
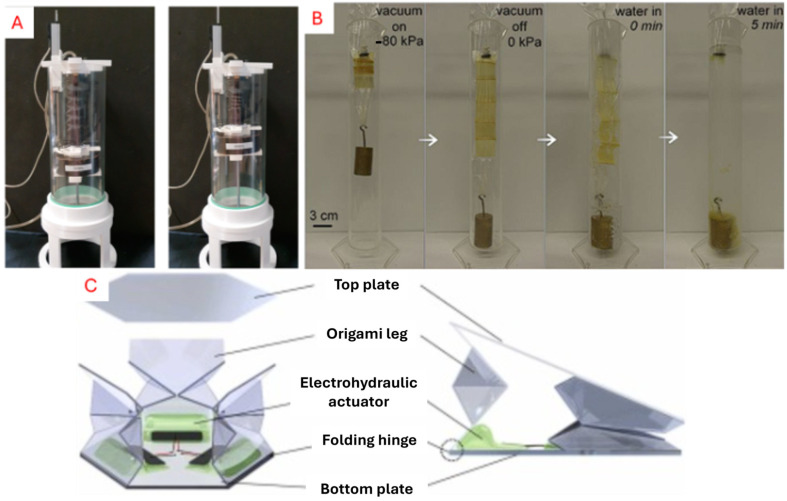
(**A**) Pneumatic automation of varying muscle-like soft robotics [131]. (**B**) Hydraulic-based muscle being actuated at different states [93]. (**C**) Muscle-like electro-hydraulic actuator in tri-legged robot [178].

We refer to fluidic actuation as the transmission of energy through a pressurised fluid medium. This implementation usually consists of a central pump or controller that connects over valves or tubes to manipulate a robot via fluid displacement through the control of pressure or flow. There are generally two media in fluidic actuation: gas and liquids [116,118,139]. Air-based actuators, or pneumatic actuators, are compressible and thus highly compliant in an actuator system [116,131]. Alternatively, the use of hydraulic actuators allows for higher force output due to the incompressible medium [93,140]. Fluid displacement enables reversible actuation folds and can facilitate cyclic actions in robot movement, making it ideal for origami patterns such as bellows, Kresling cylinders, and Miura-fold structures. A consolidation of fluidic actuators is shown in Figure 12.

#### 5.2.1. Pneumatics

Pneumatic systems are air-based actuator systems that have the capability to handle delicate tasks due to their compressible and deformable actuation. These setups are usually built with a centralised air source and chamber layout. Hence, this often results in lightweight and scalable robots. However, this also implies a weakness for high-load or accuracy tasks, as compressibility leads to nonlinear control behaviour [131,136]. Pneumatics in origami-inspired robots are more commonly found in applications that mimic muscle-like behaviour. Zaghloul and Bone [131] developed an origami-inspired soft actuator that utilises pneumatics to operate and showcased its pneumatic operation by lifting a payload mass, demonstrating the flexibility of origami robotics. Similarly, Schmitt et al. [134] developed a short pneumatic actuator with a combination of soft and hard material elements. Caiyang et al. [135] adapted a Kresling-patterned actuator instead and were able to achieve a large contraction distance with the design.

#### 5.2.2. Hydraulics

Hydraulics are fluid-based actuator systems that provide high strength and precise control, making them suitable for tasks requiring load-bearing operations. Unlike pneumatics, the liquid medium is not compressible. This introduces challenges such as overall weight, maintenance, and robustness, which hinder portability and durability. Nonetheless, hydraulics have been utilised to achieve high strength yet compactness in morphing structures. Shuguang et al. [93] developed artificial muscles based on enclosing a simple Miura-fold design principle in origami with fluidics. By adjusting the fluid volume, the origami structure was able to replicate extension and contraction behaviour via a muscle-like action.

### 5.3. Embedded Actuation

#### 5.3.1. Chemoresponsive

**Figure 13:**
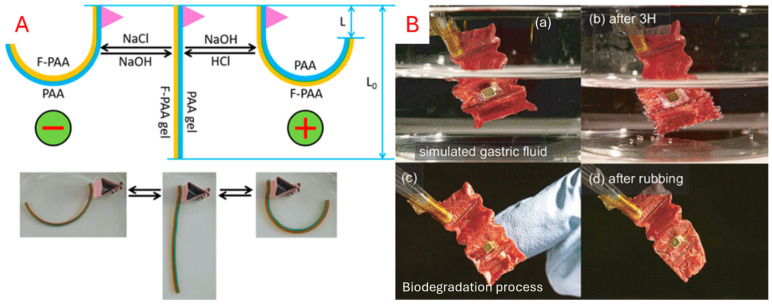
(**A**) A hydrogel-enabled robot that can change forms based on its chemical reaction [180]. (**B**) An ingestible robot for patching stomach wounds that degrades over time with stomach acid [157].

Chemoresponsive materials are those that respond to a chemical reaction, such as a change in pH level or ion concentration [179]. Hydrogels are commonly used as a chemical stimulus, where the polymer network can swell or shrink depending on pH or ionic strength. By integrating hydrogels within hinges, folding can be achieved by adjusting the pH values. This pH sensitivity can be exploited for biomedical and environmental applications. As shown in Figure 13, Geckeler et al. [141] developed a hydrogel that degrades over time with rainwater contact. When attached to a tree branch, the origami robot can release the sensor, thereby alleviating the need for sensor retrieval. Similarly, Miyashita et al. [157] created an ingestible robot for patching stomach wounds. The robot is wirelessly controlled to identify the wound and degrades over time due to stomach acid.

#### 5.3.2. Photoresponsive

**Figure 14:**
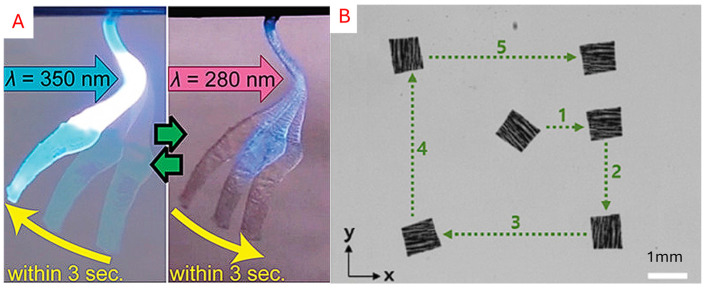
(**A**) Wavelength-adjustable hydrogel manipulation via light [149]. (**B**) Photosensitive motion of a hydrogel-infused sheet that allows for movement control [183].

Materials that deform when exposed to specific light wavelengths, such as ultraviolet (UV) or visible light, undergo molecular changes when illuminated, which often result in deformation [181]. Photoresponsive materials rely on molecules that change their composition in response to a light source. Azobenzene is a commonly used photochromic molecule that rearranges its molecules under UV light [182]. Grafting a photochromic material, such as azobenzene, into an existing polymer or LCE can induce molecular rearrangements with light exposure that can even be reversed. Alternatively, photothermal materials like carbon nanotubes can also be embedded in an SMP or polymer [148,149,181]. Heat rapidly accumulates when the material is exposed to a light source, allowing for a change in the object’s shape and molecular structure [22]. One advantage of its use case is that it can be controlled over a long, unobstructed distance with high-resolution control. As shown in Figure 14, Ikejiri et al. [149] utilised a wavelength-adjustable hydrogel to control the expansion and contraction of the hydrogel, making it suitable for artificial muscle development. Kim et al. [183] employed a bi-layered approach to enable the locomotion of a sheet of hydrogel-infused robot, allowing for positioning control. Illustration of the photoresponsive robots is presented in Figure 14.

#### 5.3.3. Electroresponsive

**Figure 15:**
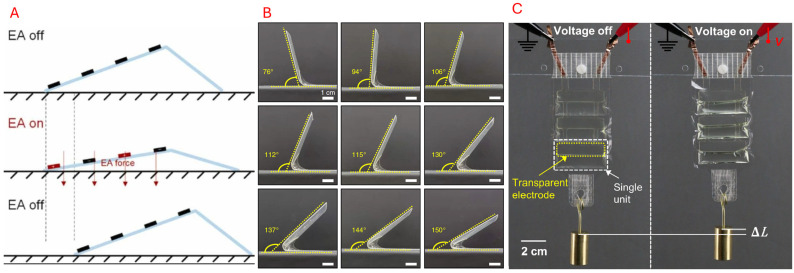
(**A**) A soft crawling-inchworm robot activated via electroadhesion [151]. (**B**) A self-folding robot controlled with varying voltages [152]. (**C**) A Peano-HASEL electrohydraulic artificial muscle that expands and contracts through high voltage [140].

A voltage or electric field can also be used on Electroactive Polymers (EAPs) to induce deformation. Dielectric elastomer (DEA) and ionic polymer actuators are two common materials used as electrical stimuli in origami-inspired robots. In DEA, a thin elastomer film is installed within two compliant electrodes [150,152]. When sufficient voltage is applied, the two electrodes undergo electrostatic attraction, squeezing and imposing a lateral expansion of the elastomer film. This expansion imitates muscle-like characteristics in origami-inspired robots, such as stretching and bending. Using dielectric fluids or electrostatic hinges can be another approach, where the hinges can be fitted with adjacent panels and controlled independently [151]. Electrical actuation is typically used for precise and rapid control and can be fine-tuned with varying angles or extensions. Xiang et al. [151] developed an inchworm robot that was activated via electroadhesion. This enabled the simple robotic structure to move forward. Similarly, by placing dielectric elastomers on hinges, Sun et al. [152] was able to develop a self-folding robot. Kellaris et al. [140] introduced Peano-HASEL electrohydraulic artificial muscles that are inextensible polymer pouches made with patterned liquid dielectric electrodes that actuate under high voltage, displacing fluids into other regions to induce expansion and contraction akin to a fluidic actuator. This actuation method reduces the reliance on a central pump or valve system, which can potentially lower costs compared to its fluidic counterparts. Figure 15 showcases some examples of electroresponsive materials.

#### 5.3.4. Magnetoresponsive

**Figure 16:**
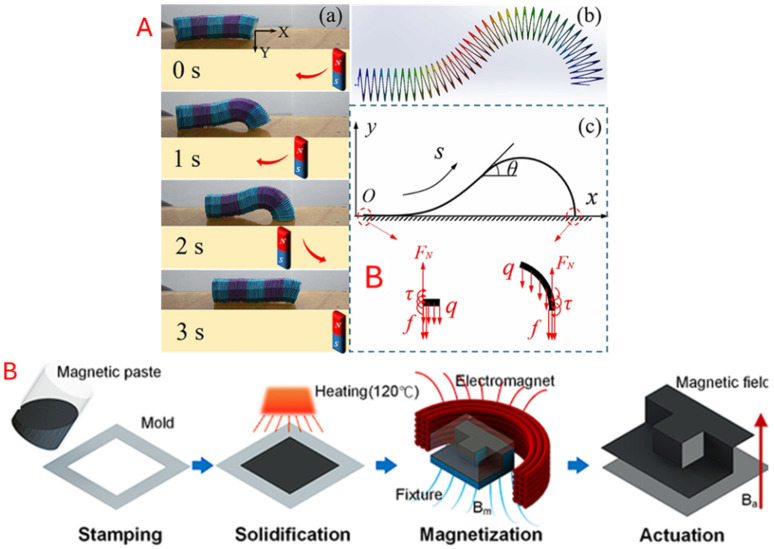
(**A**) Magnetically driven OIR worm: (**a**) actuation process, (**b**) OIR worm is modelled as a spring, and (**c**) force analysis diagram [184]. (**B**) Memory-based magnetic film to allow for repeatable actuation [40].

Magnetically charged materials can be embedded onto or within the origami structure. These magnets can then be wirelessly stimulated by other magnetic sources to incur movement or transformation in soft robotics [40,155]. Magnetic stimuli enable remote, responsive, and repeatable actuation, allowing for incredibly flexible use cases [156,157]. Tang et al. [40] developed a magnetic membrane with a memory-like property that allows magnetic wireless control by contraction of the body. Liu et al. [155] used a thermo-responsive polyvinyl chloride (PVC) film, placed between two conductive structural layers, to be activated inductively by an alternating magnetic field. The authors also used an embedded magnet on the structure to drive and direct the robot from one point to another. Figure 16 shows the magnetically responsive designs.

#### 5.3.5. Thermoresponsive

**Figure 17:**
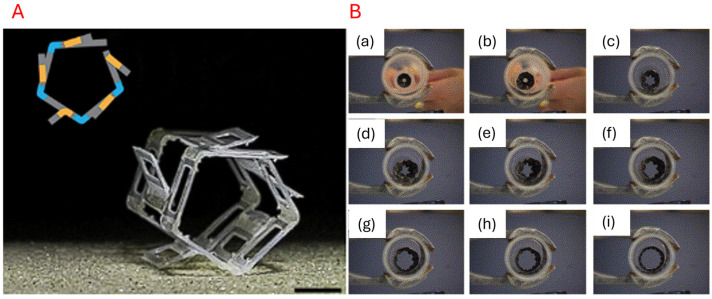
(**A**) Foldable self-propelling wheel developed with LCE materials. Its flexible design also allows for programmability [160]. (**B**) Self-expanding stent that changes size in response to body temperature. Subfigures (**a**–**i**) present the progress of the folded stent in the small tube to fully expand at above transformation temperature of 319 K [88].

Thermal stimulus materials exhibit the ability to change their shape, phase, or stiffness in response to applied heat. This functionality can be exploited through Shape Memory Alloy (SMAs) and Liquid Crystal Elastomers (LCEs). These thermally activated materials can also be programmed repeatedly, so that applying different temperatures can activate various states. Kotikian et al. [160] developed a foldable, origami-inspired robot with LCE materials, showcasing its programmable folding capabilities and the self-propelling properties of the robot (see Figure 17).

#### 5.3.6. Humidity-Responsive

**Figure 18:**
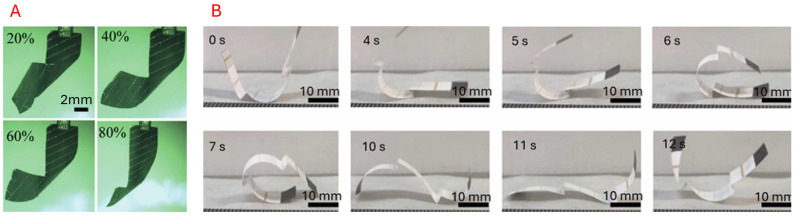
(**A**) A bi-layered humidity-sensitive actuator that is driven by varying humidity levels [161]. (**B**) A self-curling robot that is enabled via humidity [162].

Humidity-driven origami is appealing as an eco-friendly actuation method as it can be activated passively. Origami materials can be responsive to humidity or moisture levels. Hydrophilic materials expand when subjected to moisture. One can control this expansion by changing the humidity [164,185,186]. Passive actuation based on moisture can be beneficial for climate-responsive behaviour. Paper is an example of a hydrophilic material—an origami flower can “bloom” when placed in water [187]. Zuo et al. [161] used a bilayer approach with polypyrrole (PPy) and polyethylene terephthalate (PET) tape to develop a programmable actuator based on humidity changes. Mustapa et al. [162] developed a continuous rolling origami robot that curls upon exposure to constant humidity. A few examples of this actuation type are shown in Figure 18.

## 6. Application of Origami-Inspired Robots

### 6.1. Small-Scale

Origami structures have been adopted in small-scale applications ranging from micro-scale in grippers for manipulating biological specimens to millimetre- and centimetre-scale in robot locomotion.

In micro-scale, by using ultra-thin polymer or silicon sheets with crease networks in micron sizes, researchers achieved the fabrication of self-folding micro-grippers that were actuated by heat and moisture. For example, SU-8 polymer films developed with bistable Miura-Ori hinges can grasp single cells via thermal actuation, at a force in the scale of micro-newtons, with split-second response times [188]. The planar configuration is compact, which allows for easy and quick fabrication, while the creased regions supply a margin for misalignment and shock absorption. OIR micro-grippers and manipulators are useful in applications like cell sorting, minimally invasive biopsy tools [189], and automated assembly in Micro-Electro-Mechanical Systems (MEMS) [190]. The geometric transformation—from 2D to 3D—capability of origami has spurred innovations across various biomedical applications [11], such as self-folding stent grafts [88], ingestible robots for patching stomach wounds [157], and drug delivery [191]. The stringent requirements in biomedical applications require high compliance and integration, making origami-inspired implementations highly cohesive and functionally suitable.

In millimetre- and centimetre-scales, OIRs have demonstrated diverse and effective locomotion functionalities that include crawling [109,192,193], walking/wheeling [68], jumping [194], and swimming [114]. For instance, a worm-inspired robot exhibited robustness and flexibility, demonstrating compliance in peristaltic and concertina motion [109,192,193]. The versatility to adapt to various applications even extends to gripper manipulation [38,84] and human–robot interaction [195]. Origami-inspired grippers at larger scales, such as those based on the waterbomb tessellation, can lift a wide variety of objects, including delicate and heavy items, without damage [16,196]. Their ability to absorb excessive forces through structural deformation makes them particularly suitable for handling fragile and irregularly shaped objects.

Despite the tremendous advantages the OIRs can bring to these small-scale applications, there are several challenges across the board. Fabrication, especially at the micro-scale, remains the main technical challenge. High-precision microfabrication and manufacturing process control are required to avoid any variations that could damage the folding kinematics. In addition, the challenge in fabrication also arises from the limited materials, which must not only be compatible with the operating environment but also be reliable over multiple folding cycles due to the nature of the origami structure. Actuation is also a field that needs more research. As this is a small-scale application, external manipulation is impractical, while the potential active materials and embedded actuators introduce complex challenges in design, fabrication, and control strategies. The larger scales, in millimetre and centimetre sizes, although more popular, still present challenges in terms of suitable materials and fabrication techniques to bring those developed prototypes to real-world applications that require strength and reliability.

### 6.2. Medium-Scale

On the medium scale, from centimetre size, origami-inspired designs enable minimally invasive surgical tools [197,198] and comfortable wearables [199]. In wearable robotics, origami flexure joints in lightweight exoskeletons show high compliance with human joints and motion [200]. If the creases are tuned correctly, exosuits can even deliver assistive torques in gait or arm motion while remaining soft and comfortable when unpowered [201]. Origami structures are also extensively studied for their energy absorption capabilities under static and dynamic loading, up to a range of a metre [13,202]. Designs like the five-degree-of-freedom (DOF) origami with negative Poisson’s ratio properties can provide excellent energy absorption and impact resistance [203].

As the size increases, non-zero thickness accommodation will be the most challenging aspect for the origami structures. Thick materials at the folds are associated with increasing strain, potentially leading to failures such as fatigue and tearing. These bulky joints and thick facet features, designed for higher strength in medium-size applications, may also interfere with the intended performance of the overall structure. Despite numerous modelling and design methods for mechanical systems, there are no universal tools for OIRs due to their unique and creative designs, which vary from application to application. Therefore, defining the optimal thickness for OIRs remains challenging, and this challenge will increase in proportion with the size and complexity of the applications.

### 6.3. Large-Scale

Origami-inspired designs have been shown to be extremely suitable for large-scale deployable systems, where compact designs are required for stowing and a reliable unfolding method is needed for a smooth deployment [204]. Miura-Ori and cylindrical Yoshimura-based booms and solar arrays are some designs that have been used, which have demonstrated high compatibility for launches with large-area deployment in space-related tasks [205]. Extraterrestrial deployments present several challenges, including environmental durability against radiation, thermal cycling, material selection with scale-dependent reinforcements, and the design of actuators. Similarly, disaster-relief shelters that expand into habitable structures with hydraulic or manual actuation use origami tessellations with fabric-reinforced panels that showed similar compatibility features [206]. The considerations relate to the environment’s weather, ease of assembly, and a strong locking mechanism to secure creased sections.

Besides the particular challenges in each application scenario, the design and operation of origami-inspired systems at large scales introduce a distinct set of engineering challenges that must be overcome for a reliable solution in real-world applications. As size increases, the mechanical response shifts from hinge-dominated kinematics toward the facet’s strength, which is associated with phenomena such as bending, local buckling, and scale-dependent stiffness. Therefore, the selection and development of strong yet lightweight materials become key obstacles that will promote the R&D direction toward structural reinforcement, composites, and protective coating techniques to withstand harsh environments, such as UV, moisture, abrasion, thermal cycles, and radiation. To ensure a smooth deployment process, actuation and control strategies with redundant systems and embedded structural health monitoring are highlighted to facilitate safe operation.

**Figure 19:**
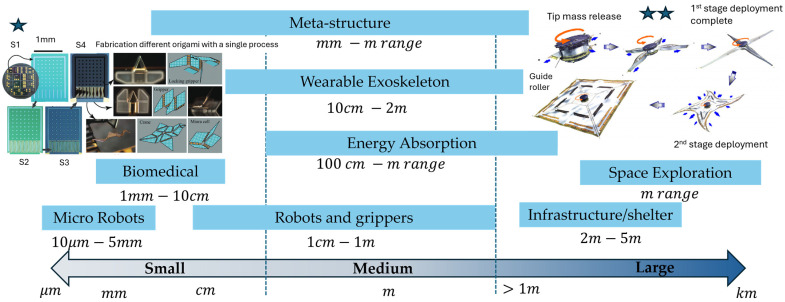
Summary of origami-inspired engineering applications at various scales. ★ Different micro-origami geometries and functions (locking gripper, gripper, crane, and Miura cell) are fabricated on the same wafer 1mm width and 21 μm thickness with four-step (S1 to S4) procedure [207]. ★★ Origami structure is adopted in 20 m-span deployable solar sail in two stages [208].

Figure 19 below summarises the applications of origami-inspired structures at various scales to emphasise the potential of this folding technique to effectively aid engineering solutions.

In summary, origami-inspired structures and principles enable compact and morphable engineering solutions across scales. However, creating a reliable design and operation system requires a unique scale-specific approach. While micro-scale systems require compatible materials and a precise fabrication process, the millimetre to centimetre range designs focus on the development and integration of active materials and advanced manufacturing techniques. At large sizes, thickness accommodation becomes a key challenge for origami structures to perform effectively, starting from the centimetre range scale. Meanwhile, extremely large applications for space exploration shift the focus to composite reinforcements and redundant deployment systems for environmental durability and safe operation.

## 7. Challenges and Outlooks

### 7.1. Reliability and Robustness

As the advantages in the motions and morphing performance of origami structures rely on their folding, their long-term robustness and reliability remain key concerns [209]. Therefore, improving fatigue resistance is one of the ongoing areas of research, especially for designs that utilise repeating operating cycles and/or bistable mechanisms [210,211,212]. In particular, soft active materials such as shape memory polymer, hydrogel, dielectric elastomer, liquid crystal elastomer, and magnetic soft materials are now one of the promising types of materials that is being developed for self-folding OIRs [22]. The soft nature of these materials raises questions about their reliability, as they are prone to common failures in operation due to fatigue, creep, and hysteresis. The development of these novel soft materials to adapt to OIRs leads to a trade-off between their mechanical strength and properties and functional performance induced by magnetic field, chemical, thermal, and electrical stimuli. For fabrication, multi-material 3D printing offers a promising solution, enabling intricate geometries and robust hard–soft coupled designs [213]. However, challenges persist, including achieving high resolution and surface finish, and ensuring robust interfacial bonding between different materials [12].

### 7.2. Complexity in Modelling and Analysis

In addition, there is also a limitation in the framework to optimise the design of origami-inspired structures that can accurately model and predict their complex behaviours, such as large deformations, rigid–flexible coupling, and various folding modes [16]. Current models often focus on rigid folding and struggle to incorporate elastic and plastic deformation or kinematic singularities [17]. Traditional rigid-linkage approaches often fail to accurately predict real-world performance, while time-consuming finite element simulations can be challenging to integrate into design iterations or real-time control. Moving forward, reduced-order models that retain essential nonlinear characteristics, paired with rapid optimisation techniques, will be critical to bridge the gap between tractable analysis and predictive accuracy. Moreover, the integration of active materials, as discussed above in the origami structure, presents another challenge level in modelling and analysis, necessitating the accommodation of the multiphysical domain. Instability is one of the most challenging problems for those soft active systems, which are receiving huge attention in research alongside the designs [214,215,216].

### 7.3. Integration with Emerging Technologies and Control Strategies

OIRs also pose a unique control challenge due to their potentially high-DOF system, the inherent nonlinearities, and the hysteresis of embedded smart materials often used for actuation. First, the nonlinear, multimodal behaviour of crease networks, including phenomena such as bistability and snap-through, makes it difficult to derive accurate analytical models for real-time control. Rigid-linkage approximations break down when panels flex, while full finite element representations are too computationally heavy for onboard controllers. As a result, conventional model-based controllers can struggle with unmodeled dynamics, leading to positioning errors or unstable folding motions. Second, the large number of DOFs inherited in an origami structure by their crease patterns and fold techniques, rather than the number of actuators, may complicate the control strategies. For example, in tendon-driven or cable-pulled systems, friction, backlash, and creep phenomenon distort the actuator–crease mapping, hence demanding advanced compensation strategies. Moreover, compliant hinge regions introduce hysteresis and time-dependent creep, which gradually affect the system’s repeatability. Lastly, many sensor techniques are hard to integrate into thin, foldable substrates. Embedding strain gauges or flex sensors along creases often increases material complexity and can interfere with fold kinematics. Vision-based feedback can track global shape but lacks the resolution to detect small-angle deviations, which are critical for precision tasks. Without robust, low-profile sensing, feedback loops remain noisy and fragile.

While most of the current OIRs rely on “on–off” control rather than feedback control, often depending on predesigned structures for specific tasks [84,100,152], only a very few feedback control strategies are observed in existing works. For example, a controllable bending motion is achieved with an embedded capacitive sensor on a two-DOF Kresling-based pneumatic-actuated robot [77], or a pressure-dependent capacitance sensor could guide an autonomous soft robot to avoid obstacles in constrained and dynamic environments [217]. The successful implementation of sensors in control strategies further promotes this research direction for the more precise control and adaptive motion of OIRs.

### 7.4. Artificial Intelligence (AI) Tools in OIRs

Recent advances in AI tools have opened a new design frontier for OIRs. Starting from the idea and design phase, AI can potentially become an inverse design tool to map the crease patterns with desired folding and morphing functions. Those tools presented in Section 2.2.5 have already involved the optimisation in which the planned patterns can be tuned by the embedded optimising algorithm (see example in Figure 6). With the increase in functions, thus the increase in features in the crease patterns, machine learning is foreseen as the promising tool to solve this issue effectively. In the prototyping and characteristic phase, an Artificial Neural Network (ANN) has been utilised to assess the fatigue and stability of the origami structure, resulting in an optimal set of design parameters with less computational costs compared with the traditional finite element method [212]. This will help to speed up the iteration design process of OIRs in general. In the operation phase, machine learning algorithms integrated into the control strategies and real-time monitoring that is being successfully applied in other fields can be adopted to the develop the OIRs toward being resilient and autonomous systems.

### 7.5. Transition from Lab Prototyping to Real-World Applications

Although many origami-inspired engineering solutions have been successfully deployed in real-world applications, many more OIRs are currently at the conceptual or laboratory stage [12,24]. In addition to the key challenges discussed in the preceding sections, we highlight that the major challenge for this transition lies in the availability of materials and their development. The material is the key factor that determines the kinematic performance, fatigue, reliability and robustness of the structure. While paper (or on a large scale, “material”) has been considered as a key factor in artistic origami [218], a similar level of research attention should be devoted to this topic to bring more OIRs from the lab to life. Many high-performance polymers and composites are difficult to recycle and may pose environmental burdens at end-of-life. Therefore, future development should also emphasise eco-friendly materials to reduce the environmental footprint of origami robots and achieve truly sustainable, circular engineering solutions.

## 8. Conclusions

Origami-inspired structures have emerged as a revolutionary design technology for robotics, offering advantages in morphology and functionality capabilities that outweigh those of traditional rigid-body designs. By encoding kinematic linkages directly into crease patterns, these foldable architectures can achieve dramatic shape changes and multi-DOF motion using minimal actuation. Their intrinsic compliance provides passive energy absorption and safe human–machine interaction, while the flat-foldable design allows for compact transport, easy deployment, and effective operation. As systematically reviewed in this work, the careful selection of fold patterns, ranging from traditional tessellation to custom crease design, is enabled by powerful computational tools, coupled with fabrication methods and integrated actuation and control. This approach enables robots that can self-deploy, comply with dynamic environments, and reconfigure on demand.

In the future, the integration of origami-inspired structures with embedded smart materials, additive manufacturing techniques, and data-driven control frameworks promises even greater levels of autonomy and versatility. Such advantages will unlock new applications that could be beyond what we can imagine, where morphology capability could evolve to perform a new function, or even to complete entire tasks. However, the research community must address key challenges in materials availability and development, control strategy for complex deformations, and enhancing long-term reliability and robustness. When tackling these challenges, a research and development approach through interdisciplinary collaboration between materials scientists, mechanisms experts, control engineers, and origami artists is needed for this field to blossom with more origami-inspired structures for the next generation of adaptable, versatile, and resilient robots.

## Figures and Tables

**Table 1:** Summary of several origami-inspired structures with their typical applications.

Design Methods	Patterns	Typical Applications	Source
Tessellations	Miura-Ori	Morphable wheel	[68]
		High-DOF robotic arm and manipulation	[31,93]
	Kresling	High-DOF motions	[67,90]
		Tunable stiffness	[39,86]
		Shock absorber	[36]
		Wave-mode converter	[94]
	Waterbomb	Deformable wheel	[71]
		Compliant Gripper	[84]
	Yoshimura	Compliant gripper	[38]
	Resch	Energy absorption	[95]
		Stiffness switching	[96]
	Honeycomb	Out-of-plane energy absorption	[29]
Vertex-centre patterns	Petal	Deformable wheel	[70]
		Deployable solar array and truss-antenna	[64]
	Flasher Pattern	Thick-panel deployable curved-surface	[65]
		Various transmission wheel	[97]
Custom designs	Origami exoskeletons	Self-reconfiguring robot with multi-tasking	[75]
	Tribot	Multigait robot	[76]
	Diamond cutouts	Multi-DOF force sensing	[85]

**Table 2:** Typical actuations in origami-inspired robots.

Category	Sub-Type	Reference
Mechanical actuation	Motors	[68,114,115]
	Tendons/cable-driven	[114,116,117,118,119,120,121,122,123,124,125]
	Bistable snap mechanism	[121,126,127,128,129,130]
Fluidic actuation	Pneumatics	[67,118,125,131,132,133,134,135,136,137]
	Hydraulics	[67,93,138,139,140]
Embedded actuation	Chemoresponsive	[141,142,143,144,145]
	Photoresponsive	[146,147,148,149]
	Electroresponsive	[76,144,150,151,152,153,154]
	Magnetoresponsive	[40,90,155,156,157]
	Thermoresponsive	[88,155,158,159,160]
	Humidity-responsive	[75,161,162,163,164]

## Data Availability

No new data were created or analysed in this study. Data sharing is not applicable to this article.

## Abbreviations

The following abbreviations are used in this manuscript:

AM	Additive Manufacturing
CFRP	Carbon Fibre Reinforced Polymer
DOF	Degree of freedom
FDM	Fused Deposition Modelling
FEM	Finite Element Method
MEMS	Micro-Electro-Mechanical Systems
OIR	Origami-Inspired Robot
OMS	Origami Multiplexed Switch
PET	Polyethylene terephthalate
PLA	Polylactic Acid
PPy	Polypyrrole
TPU	Thermoplastic polyurethane

## References

[1] Hull T. (2008). Origami. Encyclopaedia of the History of Science, Technology, and Medicine in Non-Western Cultures.

[2] Akira Y., Kiyo Y., Robert J.L. (2016). Akira Yoshizawa, Japan’s Greatest Origami Master: Featuring over 60 Models and 1000 Diagrams by the Master.

[3] Ben-Ari M. (2022). The Axioms of Origami. Mathematical Surprises.

[4] Demaine E.D., O’Rourke J. (2007). Geometric Folding Algorithms.

[5] Koryo M., Hull T. (2002). The Application of Origami Science to Map and Atlas Design. Origami 3.

[6] Lang R.J. A computational algorithm for origami design. Proceedings of the Twelfth Annual Symposium on Computational Geometry–SCG’96.

[7] Tachi T. (2010). Freeform Origami Software. https://origami.c.u-tokyo.ac.jp/~tachi/software/.

[8] Tachi T. (2011). Rigid-Foldable Thick Origami. Origami 5.

[9] Rus D., Sung C. (2018). Spotlight on origami robots. Sci. Robot..

[10] Misseroni D., Pratapa P.P., Liu K., Kresling B., Chen Y., Daraio C., Paulino G.H. (2024). Origami engineering. Nat. Rev. Methods Primers.

[11] Ahmed A.R., Gauntlett O.C., Camci-Unal G. (2021). Origami-Inspired Approaches for Biomedical Applications. ACS Omega.

[12] Xue W., Jian B., Jin L., Wang R., Ge Q. (2025). Origami Robots: Design, Actuation, and 3D Printing Methods. Adv. Mater. Technol..

[13] Xiang X., Lu G., You Z. (2020). Energy absorption of origami inspired structures and materials. Thin-Walled Struct..

[14] Hussain K.F., Cantwell W.J., Khan K.A. (2025). Review of Recent Advances in Origami-Inspired Structures for Enhanced Energy Absorption: Trends and Engineering Applications. Arab. J. Sci. Eng..

[15] Yue S. (2023). A Review of Origami-Based Deployable Structures in Aerospace Engineering. J. Phys. Conf. Ser..

[16] Meloni M., Cai J., Zhang Q., Sang-Hoon Lee D., Li M., Ma R., Parashkevov T.E., Feng J. (2021). Engineering Origami: A Comprehensive Review of Recent Applications, Design Methods, and Tools. Adv. Sci..

[17] Chen Y., Yan J., Feng J. (2019). Geometric and Kinematic Analyses and Novel Characteristics of Origami-Inspired Structures. Symmetry.

[18] Fonseca L.M., Rodrigues G.V., Savi M.A. (2022). An overview of the mechanical description of origami-inspired systems and structures. Int. J. Mech. Sci..

[19] Yan P., Huang H., Meloni M., Li B., Cai J. (2025). Mechanical Properties Inside Origami-Inspired Structures: An Overview. Appl. Mech. Rev..

[20] Peraza-Hernandez E.A., Hartl D.J., Malak R.J., Lagoudas D.C. (2014). Origami-inspired active structures: A synthesis and review. Smart Mater. Struct..

[21] Ning X., Wang X., Zhang Y., Yu X., Choi D., Zheng N., Kim D.S., Huang Y., Zhang Y., Rogers J.A. (2018). Assembly of Advanced Materials into 3D Functional Structures by Methods Inspired by Origami and Kirigami: A Review. Adv. Mater. Interfaces.

[22] Leanza S., Wu S., Sun X., Qi H.J., Zhao R.R. (2024). Active Materials for Functional Origami. Adv. Mater..

[23] Ai C., Chen Y., Xu L., Li H., Liu C., Shang F., Xia Q., Zhang S. (2021). Current Development on Origami/Kirigami-Inspired Structure of Creased Patterns toward Robotics. Adv. Eng. Mater..

[24] Rus D., Tolley M.T. (2018). Design, fabrication and control of origami robots. Nat. Rev. Mater..

[25] Song K., Li H., Li Y., Ma J., Zhou X. (2024). A review of curved crease origami: Design, analysis, and applications. Front. Phys..

[26] Gilewski W., Pełczyński J., Stawarz P. (2014). A Comparative Study of Origami Inspired Folded Plates. Procedia Eng..

[27] Robert L. Robert Lang Origami. https://langorigami.com/artworks/.

[28] Alfattani R., Lusk C. Design of a Bistable Origami Reverse-Fold Using Spherical Kinematics. Volume 5B: 41st Mechanisms and Robotics Conference. Proceedings of the ASME 2017 International Design Engineering Technical Conferences and Computers and Information in Engineering Conference.

[29] Zhai J., Zhang D., Li M., Cui C., Cai J. (2022). Out-of-plane energy absorption and crush behavior of origami honeycomb. Thin-Walled Struct..

[30] Krishnapuram S., Xiao X., Ren H. (2024). Comparative Study of Mechanical Scaling Effects of Origami-Inspired Motion Generation Mechanisms with Multi-Degree Vertices. Actuators.

[31] Barreto R.L.P., Morlin F.V., de Souza M.B., Carboni A.P., Martins D. (2021). Multiloop origami inspired spherical mechanisms. Mech. Mach. Theory.

[32] Lv C., Krishnaraju D., Konjevod G., Yu H., Jiang H. (2014). Origami based mechanical metamaterials. Sci. Rep..

[33] Schenk M., Guest S.D. (2013). Geometry of Miura-folded metamaterials. Proc. Natl. Acad. Sci. USA.

[34] Wickeler A.L., Naguib H.E. (2020). Novel origami-inspired metamaterials: Design, mechanical testing and finite element modelling. Mater. Des..

[35] Woodruff S.R., Filipov E.T. (2020). A bar and hinge model formulation for structural analysis of curved-crease origami. Int. J. Solids Struct..

[36] Masana R., Dalaq A.S., Khazaaleh S., Daqaq M.F. (2024). The Kresling origami spring: A review and assessment. Smart Mater. Struct..

[37] Filipov E., Liu K., Tachi T., Schenk M., Paulino G. (2017). Bar and hinge models for scalable analysis of origami. Int. J. Solids Struct..

[38] Xue W., Jin L., Jian B., Ge Q. (2025). Origami-Based Flexible Robotic Grippers via Hard-Soft Coupled Multimaterial 3D Printing. Soft Robot..

[39] Wo Z., Filipov E.T. (2023). Stiffening multi-stable origami tubes by outward popping of creases. Extrem. Mech. Lett..

[40] Tang D., Zhang C., Sun H., Dai H., Xie J., Fu J., Zhao P. (2021). Origami-inspired magnetic-driven soft actuators with programmable designs and multiple applications. Nano Energy.

[41] Zhu Y., Filipov E.T. (2021). Rapid multi-physics simulation for electro-thermal origami systems. Int. J. Mech. Sci..

[42] Ario I., Nakazawa M. (2010). Non-linear dynamic behaviour of multi-folding microstructure systems based on origami skill. Int. J. Non-Linear Mech..

[43] Zhang X., Karagiozova D., Lu G., Durandet Y., Wang S. (2023). Analytical and finite element analyses on axial tensile behaviour of origami bellows with polygonal cross-section. Thin-Walled Struct..

[44] Hu J., Pan H. (2024). Design of constant-force mechanisms using origami. Adv. Mech. Eng..

[45] Lang R.J. (1994). Mathematical Algorithms for Origami Design. Symmetry Cult. Sci..

[46] Demaine E.D., Tachi T. Origamizer: A Practical Algorithm for Folding Any Polyhedron. Proceedings of the 33rd International Symposium on Computational Geometry (SoCG 2017).

[47] Tachi T. (2010). Origamizing Polyhedral Surfaces. IEEE Trans. Vis. Comput. Graph..

[48] Tachi T. (2010). Freeform Rigid-Foldable Structure using Bidirectionally Flat-Foldable Planar Quadrilateral Mesh. Advances in Architectural Geometry 2010.

[49] Amanda G., Sasaki K., Erik D. (2017). Origami Simulator. https://origamisimulator.org/.

[50] Ghassaei A., Demaine E.D., Gershenfeld N. Fast, Interactive Origami Simulation Using GPU Computation. Proceedings of the 7th International Meeting on Origami in Science, Mathematics and Education (7OSME).

[51] Tomohiro T. (2010). Freeform Variations of Origami. J. Geom. Graph..

[52] Schenk M., Guest S.D. Origami Folding: A Structural Engineering Approach. Proceedings of the 5th International Conference on Origami in Science, Mathematics and Education.

[53] Sasaki K., Mitani J. (2022). Simple implementation and low computational cost simulation of curved folds based on ruling-aware triangulation. Comput. Graph..

[54] Lang R.J. Mathematical Methods in Origami Design. Proceedings of the Bridges 2009: Mathematics, Music, Art, Architecture, Culture.

[55] Miura K. (2019). Miura-ori, Basics for Designing its Folding Machines. Proc. Int. Cartogr. Assoc..

[56] Resch R.D. The topological design of sculptural and architectural systems. Proceedings of the National Computer Conference and Exposition—AFIPS’73.

[57] Kresling B. Natural twist buckling in shells: From the hawkmoth’s bellows to the deployable Kresling-pattern and cylindrical Miura-ori. Proceedings of the 6th International Conference on Computation of Spatial Structures IASS-IACM 2008: “Spanning Nano to Mega”.

[58] Yoshimura Y. On the Mechanism of Buckling of a Circular Cylindrical Shell Under Axial Compression. https://ntrs.nasa.gov/citations/19930093840.

[59] Wickeler A.L., McLellan K., Sun Y.-C., Naguib H.E. (2023). 4D printed origami-inspired accordion, Kresling and Yoshimura tubes. J. Intell. Mater. Syst. Struct..

[60] Ma J., You Z. Modelling of the Waterbomb Origami Pattern and its Applications. Volume 5B: 38th Mechanisms and Robotics Conference. Proceedings of the ASME 2014 International Design Engineering Technical Conferences and Computers and Information in Engineering Conference.

[61] Gardiner M., Aigner R., Hanlon R. Fold Mapping: Parametric Design of Origami Surfaces with Periodic Tessellations. Proceedings of the 7th Origami Science Mathematics and Education Conference.

[62] Cao H., Zhou J., Chen K., He Q., Dou Q., Liu Y. (2024). Design and Optimization of an Origami Gripper for Versatile Grasping and Manipulation. Adv. Intell. Syst..

[63] Saito K., la Fuente R.P.-D., Arimoto K., Seong Y.A., Aonuma H., Niiyama R., You Z. (2020). Earwig fan designing: Biomimetic and evolutionary biology applications. Proc. Natl. Acad. Sci. USA.

[64] Wang R., Sun J., Dai J.S. (2019). Design analysis and type synthesis of a petal-inspired space deployable-foldable mechanism. Mech. Mach. Theory.

[65] Wang S., Gao Y., Huang H., Li B., Guo H., Liu R. (2022). Design of deployable curved-surface rigid origami flashers. Mech. Mach. Theory.

[66] Wang Y., Zhao Y., Han B., Dong J., Han M., Yao J. (2025). Biomimetic Origami: Planar Single-Vertex Multi-Crease Mechanism Design and Optimization. Machines.

[67] Zhang C., Zhang Z., Peng Y., Zhang Y., An S., Wang Y., Zhai Z., Xu Y., Jiang H. (2023). Plug & play origami modules with all-purpose deformation modes. Nat. Commun..

[68] Liu J., Pang Z., Li Z., Wen G., Su Z., He J., Liu K., Jiang D., Li Z., Chen S. (2025). An origami-wheeled robot with variable width and enhanced sand walking versatility. Thin-Walled Struct..

[69] Guest S.D., Pellegrino S. Inextensional wrapping of flat membranes. Proceedings of the First International Seminar on Structural Morphology.

[70] Lee D.-Y., Jung G.-P., Sin M.-K., Ahn S.-H., Cho K.-J. Deformable wheel robot based on origami structure. Proceedings of the 2013 IEEE International Conference on Robotics and Automation (ICRA).

[71] Lee D.-Y., Kim J.-S., Kim S.-R., Koh J.-S., Cho K.-J. The Deformable Wheel Robot Using Magic-Ball Origami Structure. Volume 6B: 37th Mechanisms and Robotics Conference. Proceedings of the ASME 2013 International Design Engineering Technical Conferences and Computers and Information in Engineering Conference.

[72] Feng Y., Huang X., Qiu X. (2023). Bistable morphology analysis of the flexible single-vertex origami unit cell. Extrem. Mech. Lett..

[73] Fang H., Li S., Ji H., Wang K.W. (2017). Dynamics of a bistable Miura-origami structure. Phys. Rev. E.

[74] Li J., Zhang H., Long J., Lu G. (2025). Design and analysis of quadruple Waterbomb origami with multi-stability. Extrem. Mech. Lett..

[75] Miyashita S., Guitron S., Li S., Rus D. (2017). Robotic metamorphosis by origami exoskeletons. Sci. Robot..

[76] Zhakypov Z., Paik J. (2018). Design Methodology for Constructing Multimaterial Origami Robots and Machines. IEEE Trans. Robot..

[77] Hanson N., Mensah I.A., Roberts S.F., Healey J., Wu C., Dorsey K.L. (2024). Controlling the fold: Proprioceptive feedback in a soft origami robot. Front. Robot. AI.

[78] Onal C.D., Wood R.J., Rus D. Towards printable robotics: Origami-inspired planar fabrication of three-dimensional mechanisms. Proceedings of the 2011 IEEE International Conference on Robotics and Automation.

[79] Sun J., Zhang S., Deng J., Li J., Zhou D., Wang D., Liu J., Chen W., Liu Y. (2025). High-Performance Twisted Nylon Actuators for Soft Robots. Research.

[80] Vo V.T.K., Ang M.H., Koh S.J.A. (2021). Maximal Performance of an Antagonistically Coupled Dielectric Elastomer Actuator System. Soft Robot..

[81] Nugraha M.I., Rossiter J., Conn A.T. (2025). A Low Cost Multi Degree of Freedom Capacitive Soft Sensor. IEEE Robot. Autom. Lett..

[82] Mun H., Cortes D.S.D., Youn J.-H., Kyung K.-U. (2024). Multi-Degree-of-Freedom Force Sensor Incorporated into Soft Robotic Gripper for Improved Grasping Stability. Soft Robot..

[83] Rana T., Islam S., Rahman A. (2025). Human-Centered Sensor Technologies for Soft Robotic Grippers: A Comprehensive Review. Sensors.

[84] Mathew B.P., Devasia F., Asok A., Jayadevu P., Baby R. (2022). Implementation of an origami inspired gripper robot for picking objects of variable geometry. Mater. Today Proc..

[85] Yue W., Qi J., Song X., Fan S., Fortino G., Chen C.-H., Xu C., Ren H. (2022). Origami-Inspired Structure with Pneumatic-Induced Variable Stiffness for Multi-DOF Force-Sensing. Sensors.

[86] Wang X., Qu H., Li X., Kuang Y., Wang H., Guo S., Sharma P. (2023). Multi-triangles cylindrical origami and inspired metamaterials with tunable stiffness and stretchable robotic arm. PNAS Nexus.

[87] Zhu Y., Filipov E.T. (2024). Large-scale modular and uniformly thick origami-inspired adaptable and load-carrying structures. Nat. Commun..

[88] Kuribayashi K., Tsuchiya K., You Z., Tomus D., Umemoto M., Ito T., Sasaki M. (2006). Self-deployable origami stent grafts as a biomedical application of Ni-rich TiNi shape memory alloy foil. Mater. Sci. Eng. A.

[89] Lee D.-Y., Kim J.-K., Sohn C.-Y., Heo J.-M., Cho K.-J. (2021). High–load capacity origami transformable wheel. Sci. Robot..

[90] Wu S., Ze Q., Dai J., Udipi N., Paulino G.H., Zhao R. (2021). Stretchable origami robotic arm with omnidirectional bending and twisting. Proc. Natl. Acad. Sci. USA.

[91] Zhang Z., Fan W., Long Y., Dai J., Luo J., Tang S., Lu Q., Wang X., Wang H., Chen G. (2024). Hybrid-Driven Origami Gripper with Variable Stiffness and Finger Length. Cyborg Bionic Syst..

[92] Gage T. MODeL-T Explores Origami-Inspired Foldable Flat Optics for Compact Lidar Missions; NASA’s Earth Science Technology Office. 10 February 2025. https://esto.nasa.gov/model-t-explores-origami-inspired-foldable-flat-optics-for-compact-lidar-missions/.

[93] Li S., Vogt D.M., Rus D., Wood R.J. (2017). Fluid-driven origami-inspired artificial muscles. Proc. Natl. Acad. Sci. USA.

[94] Xu Z.-L., Wang D.-F., Tachi T., Chuang K.-C. (2022). An origami longitudinal–torsional wave converter. Extrem. Mech. Lett..

[95] Chen Z., Wu T., Nian G., Shan Y., Liang X., Jiang H., Qu S. (2018). Ron Resch Origami Pattern Inspired Energy Absorption Structures. J. Appl. Mech..

[96] Jayawardana W.M.A., Elder T., Twohig T., Croll A.B. (2024). Switchable origami adhesives. Soft Matter.

[97] Felton S.M., Lee D.-Y., Cho K.-J., Wood R.J. A passive, origami-inspired, continuously variable transmission. Proceedings of the 2014 IEEE International Conference on Robotics and Automation (ICRA).

[98] Fang H., Zhang Y., Wang K.W. (2017). Origami-based earthworm-like locomotion robots. Bioinspir. Biomim..

[99] Zhao Y., Hua M., Yan Y., Wu S., Alsaid Y., He X. (2022). Stimuli-Responsive Polymers for Soft Robotics. Annu. Rev. Control Robot. Auton. Syst..

[100] Yan W., Li S., Deguchi M., Zheng Z., Rus D., Mehta A. (2023). Origami-based integration of robots that sense, decide, and respond. Nat. Commun..

[101] Qu C., Hu J., Liu X., Li Z., Ding Y. (2017). Morphology and mechanical properties of polyimide films: The effects of UV irradiation on microscale surface. Materials.

[102] Xiao P., He X., Lu Q. (2025). Exceptionally High-Temperature-Resistant Kapton-Type Polyimides with *T*_g_ > 520 °C: Synthesis via Incorporation of Spirobis(indene)-bis(benzoxazole)-Containing Diamines. Polymers.

[103] A Robertson M., Kara O.C., Paik J. (2021). Soft pneumatic actuator-driven origami-inspired modular robotic “pneumagami”. Int. J. Robot. Res..

[104] Wang D.H., Tan L.-S. (2019). Origami-Inspired Fabrication: Self-Folding or Self-Unfolding of Cross-Linked-Polyimide Objects in Extremely Hot Ambience. ACS Macro Lett..

[105] Zhu R., Fan D., Wu W., He C., Xu G., Dai J.S., Wang H. (2023). Soft Robots for Cluttered Environments Based on Origami Anisotropic Stiffness Structure (OASS) Inspired by Desert Iguana. Adv. Intell. Syst..

[106] Wagner M.A., Huang J.-L., Okle P., Paik J., Spolenak R. (2020). Hinges for origami-inspired structures by multimaterial additive manufacturing. Mater. Des..

[107] Lazarus N., Smith G.L., Dickey M.D. (2019). Self-Folding Metal Origami. Adv. Intell. Syst..

[108] Onal C.D., Wood R.J., Rus D. (2012). An origami-inspired approach to worm robots. IEEE/ASME Trans. Mechatron..

[109] Koh J.-S., Cho K.-J. (2013). Omega-Shaped Inchworm-Inspired Crawling Robot with Large-Index-and-Pitch (LIP) SMA Spring Actuators. IEEE/ASME Trans. Mechatron..

[110] Xiang X., Lu G., Ruan D., You Z., Zolghadr M. (2017). Large deformation of an arc-Miura structure under quasi-static load. Compos. Struct..

[111] An B., Miyashita S., Ong A., Tolley M.T., Demaine M.L., Demaine E.D., Wood R.J., Rus D. (2018). An End-to-End Approach to Self-Folding Origami Structures. IEEE Trans. Robot..

[112] Chen Y., Shi P., Bai Y., Li J., Feng J., Sareh P. (2023). Engineered origami crease perforations for optimal mechanical performance and fatigue life. Thin-Walled Struct..

[113] Chen Y., Shi P., Bai Y., Li J., Feng J., Sareh P. (2024). Effects of perforated creases on the mechanical behavior and fatigue life of thick origami structures. Mech. Based Des. Struct. Mach..

[114] Yang Z., Chen D., Levine D.J., Sung C. (2021). Origami-Inspired Robot That Swims via Jet Propulsion. IEEE Robot. Autom. Lett..

[115] Ze Q., Wu S., Dai J., Leanza S., Ikeda G., Yang P.C., Iaccarino G., Zhao R.R. (2022). Spinning-enabled wireless amphibious origami millirobot. Nat. Commun..

[116] Lee S., Cha Y. (2025). Analysis of Kresling origami bellows for pneumatic pump. Sens. Actuators A Phys..

[117] Liu M., Wang C., Shi C., Guo H., Liu R. (2025). Bio-inspired deployable cable-driven origami gripper with variable finger length for space capture. Aerosp. Sci. Technol..

[118] Zhang Z., Chen G., Wu H., Kong L., Wang H. (2020). A Pneumatic/Cable-driven Hybrid Linear Actuator with Combined Structure of Origami Chambers and Deployable Mechanism. IEEE Robot. Autom. Lett..

[119] Kim J., Im E., Lee Y., Cha Y. (2024). Quadrupedal robot with tendon-driven origami legs. Sens. Actuators A Phys..

[120] Salerno M., Zhang K., Menciassi A., Dai J.S. (2016). A Novel 4-DOF Origami Grasper with an SMA-Actuation System for Minimally Invasive Surgery. IEEE Trans. Robot..

[121] Liu W., Bai X., Yang H., Bao R., Liu J. (2024). Tendon Driven Bistable Origami Flexible Gripper for High-Speed Adaptive Grasping. IEEE Robot. Autom. Lett..

[122] Kim S.-J., Lee D.-Y., Jung G.-P., Cho K.-J. (2018). An origami-inspired, self-locking robotic arm that can be folded flat. Sci. Robot..

[123] Hoff E.V., Jeong D., Lee K. OrigamiBot-I: A thread-actuated origami robot for manipulation and locomotion. Proceedings of the 2014 IEEE/RSJ International Conference on Intelligent Robots and Systems.

[124] Lee K., Wang Y., Zheng C. (2020). TWISTER Hand: Underactuated Robotic Gripper Inspired by Origami Twisted Tower. IEEE Trans. Robot..

[125] Wang J., Yang H., Zhang J., Liu H., Zhong Y., Hu Y., Liu Y., Xia C., Wu J. (2025). Versatile Rigid-Flexible Coupling Modules: Enhancing Soft Origami Structures with Cable-Driven Parallel Mechanisms. Adv. Intell. Syst..

[126] Yeow B.S., Cai C.J., Kalairaj M.S., Hoo F.W., Lee Z.X., Tan J.C.S., Ho J.R., Ma V.M., Huang H., Ren H. Origami-Inspired Snap-through Bistability in Parallel and Curved Mechanisms Through the Inflection of Degree Four Vertexes. Proceedings of the 2021 IEEE International Conference on Robotics and Automation (ICRA).

[127] Zhang L., Pan F., Ma Y., Yang K., Guo S., Chen Y. (2023). Bistable reconfigurable origami metamaterials with high load-bearing and low state-switching forces. Extrem. Mech. Lett..

[128] Morgan M.R., Lang R.J., Magleby S.P., Howell L.L. (2016). Towards developing product applications of thick origami using the offset panel technique. Mech. Sci..

[129] Buscicchio A., Alessandrino G., Troise A., Sironi T., Gloder A. SolarCube: An Origami-Inspired Lightweight Deployable Solar Panel for Nanosatellites. Proceedings of the 2023 13th European Space Power Conference (ESPC).

[130] Yan W., Mehta A. A crawling robot driven by a folded self-sustained oscillator. Proceedings of the 2022 IEEE 5th International Conference on Soft Robotics (RoboSoft).

[131] Zaghloul A., Bone G.M. (2023). Origami-Inspired Soft Pneumatic Actuators: Generalization and Design Optimization. Actuators.

[132] Liu J., Ma G., Ma Z., Zuo S. (2023). Origami-inspired soft-rigid hybrid contraction actuator and its application in pipe-crawling robot. Smart Mater. Struct..

[133] Yoon C., Xiao R., Park J., Cha J., Nguyen T.D., Gracias D.H. (2014). Functional stimuli responsive hydrogel devices by self-folding. Smart Mater. Struct..

[134] Schmitt F., Piccin O., Barbé L., Bayle B. An Origami-Inspired Flexible Pneumatic Actuator. Proceedings of the 2018 IEEE/RSJ International Conference on Intelligent Robots and Systems (IROS).

[135] Caiyang E., Wang B., Guo Z., Zhang H., Xu Q., Chen J. (2025). An origami-inspired 3D-printed soft foldable actuator with large contraction deformation and strong actuation capability. Smart Mater. Struct..

[136] Martinez R., Fish C., Chen X., Whitesides G.M. (2012). Elastomeric Origami: Programmable Paper-Elastomer Composites as Pneumatic Actuators. Adv. Funct. Mater..

[137] Yi J., Chen X., Song C., Wang Z. (2018). Fiber-Reinforced Origamic Robotic Actuator. Soft Robot..

[138] Kim S., Kang J., Yoo S., Cha Y. (2025). Soft origami tripod based on electrohydraulic actuator for twisting motion. Sens. Actuators A Phys..

[139] Onorati S., Semproni F., Paternò L., Casagrande G., Iacovacci V., Menciassi A. A hydraulic soft robotic detrusor based on an origami design. Proceedings of the 2023 IEEE International Conference on Robotics and Automation (ICRA).

[140] Kellaris N., Venkata V.G., Smith G.M., Mitchell S.K., Keplinger C. (2018). Peano-HASEL actuators: Muscle-mimetic, electrohydraulic transducers that linearly contract on activation. Sci. Robot..

[141] Geckeler C., Pizzani B.A., Mintchev S. Biodegradable Origami Gripper Actuated with Gelatin Hydrogel for Aerial Sensor Attachment to Tree Branches. Proceedings of the 2023 IEEE International Conference on Robotics and Automation (ICRA).

[142] Huang Z., Wei C., Dong L., Wang A., Yao H., Guo Z., Mi S. (2022). Fluid-driven hydrogel actuators with an origami structure. iScience.

[143] Ionov L. (2013). 3D microfabrication using stimuli-responsive self-folding polymer films. Polym. Rev..

[144] Gracias D.H. (2013). Stimuli responsive self-folding using thin polymer films. Curr. Opin. Chem. Eng..

[145] Huang H.-W., Tibbitt M.W., Huang T.-Y., Nelson B.J. (2019). Matryoshka-Inspired Micro-Origami Capsules to Enhance Loading, Encapsulation, and Transport of Drugs. Soft Robot..

[146] Aggarwal A., Li C., Stupp S.I., de la Cruz M.O. (2022). Controlling the Shape Morphology of Origami-Inspired Photoresponsive Hydrogels. Soft Matter.

[147] Li C., Xue Y., Han M., Palmer L.C., Rogers J.A., Huang Y., Stupp S.I. (2021). Synergistic photoactuation of bilayered spiropyran hydrogels for predictable origami-like shape change. Matter.

[148] Patel H., Chen J., Hu Y., Erturk A. (2022). Photo-responsive hydrogel-based re-programmable metamaterials. Sci. Rep..

[149] Ikejiri S., Takashima Y., Osaki M., Yamaguchi H., Harada A. (2018). Solvent-Free Photoresponsive Artificial Muscles Rapidly Driven by Molecular Machines. J. Am. Chem. Soc..

[150] Li Y., Zhang T. (2022). Modeling and Characterizing Two Dielectric Elastomer Folding Actuators for Origami-Inspired Robot. IEEE Robot. Autom. Lett..

[151] Xiang C., Sun H., Wu T., Wu B., Guan Y., Zou T. (2024). Electroadhesion-driven crawling robots based on origami mechanism. Sens. Actuators A Phys..

[152] Sun Y., Li D., Wu M., Yang Y., Su J., Wong T., Xu K., Li Y., Li L., Yu X. (2022). Origami-inspired folding assembly of dielectric elastomers for programmable soft robots. Microsyst. Nanoeng..

[153] Kirkman S., Rothemund P., Acome E., Keplinger C. (2021). Electromechanics of planar HASEL actuators. Extrem. Mech. Lett..

[154] Rothemund P., Kellaris N., Mitchell S.K., Acome E., Keplinger C. (2020). HASEL Artificial Muscles for a New Generation of Lifelike Robots—Recent Progress and Future Opportunities. Adv. Mater..

[155] Liu J., Chen X., Lahondes Q., Esendag K., Damian D., Miyashita S. Origami Robot Self-folding by Magnetic Induction. Proceedings of the 2022 IEEE/RSJ International Conference on Intelligent Robots and Systems (IROS).

[156] These origami robots could one day deliver drugs inside your body. AAAS Articles DO Group. March 2021. https://www.science.org/content/article/these-origami-robots-could-one-day-deliver-drugs-inside-your-body.

[157] Miyashita S., Guitron S., Yoshida K., Li S., Damian D.D., Rus D. Ingestible, controllable, and degradable origami robot for patching stomach wounds. Proceedings of the 2016 IEEE International Conference on Robotics and Automation (ICRA).

[158] Yuk H., Kim D., Lee H., Jo S., Shin J.H. (2011). Shape memory alloy-based small crawling robots inspired by C. elegans. Bioinspir. Biomim..

[159] Na J., Evans A.A., Bae J., Chiappelli M.C., Santangelo C.D., Lang R.J., Hull T.C., Hayward R.C. (2015). Programming Reversibly Self-Folding Origami with Micropatterned Photo-Crosslinkable Polymer Trilayers. Adv. Mater..

[160] Kotikian A., McMahan C., Davidson E.C., Muhammad J.M., Weeks R.D., Daraio C., Lewis J.A. (2019). Untethered soft robotic matter with passive control of shape morphing and propulsion. Sci. Robot..

[161] Zuo J., Chen H., Gu J., Zhang W., Zhang Z., Huang G. (2023). A facile method for fabricating humidity-sensitive bilayer actuators with programmable deformation. Sens. Actuators A Phys..

[162] Mustapa A., Abdullah A.H., Idris W.F.W., Ismail Z. (2025). Pencil yourself a humidity-driven continuous roll Paperbot. Sens. Actuators B Chem..

[163] Chen Z., Li Y., Li Q. (2021). Hydrogel-driven origami metamaterials for tunable swelling behavior. Mater. Des..

[164] Okuzaki H., Kuwabara T., Funasaka K., Saido T. (2013). Humidity-Sensitive Polypyrrole Films for Electro-Active Polymer Actuators. Adv. Funct. Mater..

[165] Trivedi D., Rahn C.D., Kier W.M., Walker I.D. (2008). Soft Robotics: Biological Inspiration, State of the Art, and Future Research. Appl. Bionics Biomech..

[166] Pujada-Gamarra E., Lavayen-Farfán D., Olivera-Oliva D., Rodríguez-Hernández J. (2025). Origami-Inspired Photovoltaic Modules—Development of Ecofriendly Solutions for Naval and Mining Operations. Eng. Proc..

[167] Blumenschein L.H., Coad M.M., Haggerty D.A., Okamura A.M., Hawkes E.W. (2020). Design, Modeling, Control, and Application of Everting Vine Robots. Front. Robot. AI.

[168] Stokes A.A., Shepherd R.F., Morin S.A., Ilievski F., Whitesides G.M. (2014). A Hybrid Combining Hard and Soft Robots. Soft Robot..

[169] Bellinger A.M., Jafari M., Grant T.M., Zhang S., Slater H.C., Wenger E.A., Mo S., Lee Y.-A.L., Mazdiyasni H., Kogan L. (2016). Oral, ultra–long-lasting drug delivery: Application toward malaria elimination goals. Sci. Transl. Med..

[170] Seyidoğlu B., Rafsanjani A. (2024). A textile origami snake robot for rectilinear locomotion. Device.

[171] Zhakypov Z., Falahi M., Shah M., Paik J. The design and control of the multi-modal locomotion origami robot, Tribot. Proceedings of the 2015 IEEE/RSJ International Conference on Intelligent Robots and Systems (IROS).

[172] Hu Q., Chen Z., Dong E., Sun D. (2025). Design and Control of a Modular, Untethered Soft Origami Robot Driven by SMA Coils. IEEE Trans. Ind. Electron..

[173] Yang D., Mishra S., Aukes D.M., Zhang W. Design, Planning, and Control of an Origami-inspired Foldable Quadrotor. Proceedings of the 2019 American Control Conference (ACC).

[174] Hu J., Li H., Chen W. (2021). A squid-inspired swimming robot using folding of origami. J. Eng..

[175] Hu B., Zhou Z., Qu H., Hu H., Wang X., Wang H., Liu W., Guo S. (2025). Kirigami-based soft actuators and tendon-driven robots. Sens. Actuators A Phys..

[176] Jiang Y., Li Y., Liu K., Zhang H., Tong X., Chen D., Wang L., Paik J. (2023). Ultra-tunable bistable structures for universal robotic applications. Cell Rep. Phys. Sci..

[177] Zhang Z., Chen D., Wu H., Bao Y., Chai G. (2016). Non-contact magnetic driving bioinspired Venus flytrap robot based on bistable anti-symmetric CFRP structure. Compos. Struct..

[178] Kang J., Kim S., Cha Y. (2024). Soft origami tripod based on electrohydraulic actuator for multimodal motions. Sens. Actuators A Phys..

[179] Gao K., Xu K. (2025). Advancements and Prospects of pH-Responsive Hydrogels in Biomedicine. Gels.

[180] Zhao L., Huang J., Zhang Y., Wang T., Sun W., Tong Z. (2017). Programmable and Bidirectional Bending of Soft Actuators Based on Janus Structure with Sticky Tough PAA-Clay Hydrogel. ACS Appl. Mater. Interfaces.

[181] Ding M., Jing L., Yang H., Machnicki C., Fu X., Li K., Wong I., Chen P.-Y. (2020). Multifunctional soft machines based on stimuli-responsive hydrogels: From freestanding hydrogels to smart integrated systems. Mater. Today Adv..

[182] Derkowska-Zielińska B., Guerchi A., Kowalska D., Smokal V., Czaplicki R. New azobenzene-containing light-responsive polymers for soft robotics. Proceedings of the Soft Mechatronics and Wearable Systems 2025 (Volume PC13434).

[183] Kim D., Lee H., Kwon S., Sung Y.J., Song W.K., Park S. (2020). Bilayer Hydrogel Sheet-Type Intraocular Microrobot for Drug Delivery and Magnetic Nanoparticles Retrieval. Adv. Health Mater..

[184] Jin Y., Li J., Liu S., Cao G., Liu J. (2023). A worm-inspired robot based on origami structures driven by the magnetic field. Bioinspir. Biomim..

[185] Okuzaki H., Kunugi T. (1997). Adsorption-induced chemomechanical behavior of polypyrrole films. J. Appl. Polym. Sci..

[186] Okuzaki H., Funasaka K. (1999). Electrically Driven Polypyrrole Film Actuator Working in Air. J. Intell. Mater. Syst. Struct..

[187] Just Add Water—Blooming Flower Trick—YouTube. https://www.youtube.com/watch?v=DuhdLonimdU.

[188] Mackay R.E., Le H.R., Keatch R.P. (2011). Design optimisation and fabrication of SU-8 based electro-thermal micro-grippers. J. Micro-Nano Mechatron..

[189] Feng B., Liu Y., Zhang J., Qu S., Yang W. (2025). Miniature origami robot for various biological micromanipulations. Nat. Commun..

[190] Kim K., Nilsen E., Huang T., Kim A., Ellis M., Skidmore G., Lee J.-B. (2004). Metallic microgripper with SU-8 adaptor as end-effectors for heterogeneous micro/nano assembly applications. Microsyst. Technol..

[191] Huang H., Zhang H., Du N., Lyu Y., Xu J., Fu H., Guan Y., Nan K. (2025). Drug origami: A computation-guided approach for customizable drug release kinetics of oral formulations. Matter.

[192] Ze Q., Wu S., Nishikawa J., Dai J., Sun Y., Leanza S., Zemelka C., Novelino L.S., Paulino G.H., Zhao R.R. (2022). Soft robotic origami crawler. Sci. Adv..

[193] Pagano A., Yan T., Chien B., Wissa A., Tawfick S. (2017). A crawling robot driven by multi-stable origami. Smart Mater. Struct..

[194] Sadeghi S., Betsill B.D., Li S. Design and Optimization of an Origami-Inspired Jumping Mechanism With Nonlinear Stiffness Properties. Volume 8: 31st Conference on Mechanical Vibration and Noise. Proceedings of the ASME 2019 International Design Engineering Technical Conferences and Computers and Information in Engineering Conference.

[195] Hedayati H., Suzuki R., Rees W., Leithinger D., Szafir D. (2022). Designing Expandable-Structure Robots for Human-Robot Interaction. Front. Robot. AI.

[196] Tang J., Wei F. (2021). Miniaturized Origami Robots: Actuation Approaches and Potential Applications. Macromol. Mater. Eng..

[197] Suzuki H., Wood R.J. (2020). Origami-inspired miniature manipulator for teleoperated microsurgery. Nat. Mach. Intell..

[198] Yang Y., Vella K., Holmes D.P. Grasping with Kirigami Shells. https://www.science.org/doi/10.1126/scirobotics.abd6426.

[199] Li D., Yumbla E.Q., Olivas A., Sugar T., Ben Amor H., Lee H., Zhang W., Aukes D.M. (2023). Origami-Inspired Wearable Robot for Trunk Support. IEEE/ASME Trans. Mechatron..

[200] Carmona D., Xun Q., Jia X., Ren H. A Novel Wearable Origami Device for Head and Cervical Spine Protection in Falls. Proceedings of the 2022 2nd Asian Conference on Innovation in Technology (ASIANCON).

[201] Chen Z., Tighe B., Zhao J. (2022). Origami-Inspired Modules Enable A Reconfigurable Robot with Programmable Shapes and Motions. IEEE/ASME Trans. Mechatron..

[202] Wu J., Zhang Y., Li K., Su L. (2022). Origami-inspired metamaterials hierarchical structure with tailorable crushing behavior. Constr. Build. Mater..

[203] Chen B., Ni X., Zhou L., Zi B., Li E., Zhang D. (2025). Development of Bioinspired Five-DOF Origami for Robotic Spine Assistive Exoskeleton. IEEE Trans. Robot..

[204] Nohooji H.R., Voos H. (2025). Compliant Robotics in Space: A Prospective Review of Soft and Deformable Systems for Space Missions. Adv. Intell. Syst..

[205] Jasim B., Taheri P. An Origami-Based Portable Solar Panel System. Proceedings of the 2018 IEEE 9th Annual Information Technology, Electronics and Mobile Communication Conference (IEMCON).

[206] Verzoni A., Rais-Rohani M. (2022). Transition analysis of flat-foldable origami-inspired deployable shelter concepts. Eng. Struct..

[207] Zhu Y., Birla M., Oldham K.R., Filipov E.T. (2020). Elastically and Plastically Foldable Electrothermal Micro-Origami for Controllable and Rapid Shape Morphing. Adv. Funct. Mater..

[208] Tsuda Y., Mori O., Funase R., Sawada H., Yamamoto T., Saiki T., Endo T., Kawaguchi J. (2011). Flight status of IKAROS deep space solar sail demonstrator. Acta Astronaut..

[209] Moshtaghzadeh M., Izadpanahi E., Mardanpour P. (2022). Prediction of fatigue life of a flexible foldable origami antenna with Kresling pattern. Eng. Struct..

[210] Fakhari H.E., Eslami H., Moshtaghzadeh M., Mardanpour P. (2024). A comprehensive study of a new cylindrical flexible Miura-Ori origami: Kinematics, FEA, and fatigue assessments. Aerosp. Sci. Technol..

[211] Moshtaghzadeh M., Bakhtiari A., Izadpanahi E., Mardanpour P. Stability and Fatigue Analysis of an Adaptive Origami Antenna Structure with Kresling Pattern. Proceedings of the AIAA SCITECH 2022 Forum.

[212] Moshtaghzadeh M., Bakhtiari A., Mardanpour P. (2022). Artificial Neural Network-based Finite Element method for assessing fatigue and stability of an origami-inspired structure. Eng. Struct..

[213] Son H., Park Y., Na Y., Yoon C. (2022). 4D Multiscale Origami Soft Robots: A Review. Polymers.

[214] Mathew A.T., Vo T.V.K., Koh S.J.A. (2019). A molecular perspective to analytical modeling that reveals new instabilities in dielectric elastomer transducers. J. Mech. Phys. Solids.

[215] Arora N., Chen V., Cherkasov A., Xiang Y., Juhl A., Buskohl P., Rudykh S. (2024). Magnetically-Programmed Instability-Driven Pattern Transformations in Soft Materials. Adv. Funct. Mater..

[216] Cui Y., Hu J., Dong Z., Li B., Chang C. (2025). Temperature-triggered inflatable hydrogel muscles with snap-through instability for untethered robots. Nat. Commun..

[217] Chen S., Yu Z., Duan A., Tongne A., Li Y., Zhang W. (2025). A Kresling origami-enabled soft robot toward autonomous obstacle avoidance and wall-climbing. Sens. Actuators B Chem..

[218] Robert J.L. Paper. Robert Lang Origami. 9 August 2011. https://langorigami.com/article/paper/.

